# Ethanol Extraction of Polar Lipids from *Nannochloropsis oceanica* for Food, Feed, and Biotechnology Applications Evaluated Using Lipidomic Approaches

**DOI:** 10.3390/md19110593

**Published:** 2021-10-21

**Authors:** Tânia Melo, Ana R. P. Figueiredo, Elisabete da Costa, Daniela Couto, Joana Silva, M. Rosário Domingues, Pedro Domingues

**Affiliations:** 1 Mass Spectrometry Center, LAQV-REQUIMTE, Department of Chemistry, University of Aveiro, Santiago University Campus, 3810-193 Aveiro, Portugal; ana90@ua.pt (A.R.P.F.); elisabetecosta@ua.pt (E.d.C.); danielacouto@ua.pt (D.C.); mrd@ua.pt (M.R.D.); 2 CESAM-Centre for Environmental and Marine Studies, Department of Chemistry, University of Aveiro, Santiago University Campus, 3810-193 Aveiro, Portugal; 3 Allmicroalgae—Natural Products S.A., Avenida das Forças Armadas, 125, 7º Piso, 1600-079 Lisboa, Portugal; joana.g.silva@allmicroalgae.com

**Keywords:** *Nannochloropsis oceanica*, solvent extraction, lipidomics, glycolipids, phospholipids, betaine lipids

## Abstract

*Nannochloropsis oceanica* can accumulate lipids and is a good source of polar lipids, which are emerging as new value-added compounds with high commercial value for the food, nutraceutical, and pharmaceutical industries. Some applications may limit the extraction solvents, such as food applications that require safe food-grade solvents, such as ethanol. However, the effect of using ethanol as an extraction solvent on the quality of the extracted polar lipidome, compared to other more traditional methods, is not yet well established. In this study, the polar lipid profile of *N. oceanica* extracts was obtained using different solvents, including chloroform/methanol (CM), dichloromethane/methanol (DM), dichloromethane/ethanol (DE), and ethanol (E), and evaluated by modern lipidomic methods using LC-MS/MS. Ultrasonic bath (E + USB)- and ultrasonic probe (E + USP)-assisted methodologies were implemented to increase the lipid extraction yields using ethanol. The polar lipid signature and antioxidant activity of DM, E + USB, and E + USP resemble conventional CM, demonstrating a similar extraction efficiency, while the DE and ethanol extracts were significantly different. Our results showed the impact of different extraction solvents in the polar lipid composition of the final extracts and demonstrated the feasibility of E + USB and E + USP as safe and food-grade sources of polar lipids, with the potential for high-added-value biotechnological applications.

## 1. Introduction

Microalgae are emerging as a valuable source of lipids for biotechnological applications. In particular, the marine oleaginous microalgae *Nannochloropsis oceanica* is of great interest because it can produce and accumulate lipids. Although the raw biomass of *N. oceanica* is still not approved for human consumption, this microalga can produce higher amounts of omega-3 polyunsaturated fatty acids (PUFAs), such as eicosapentaenoic acid (EPA), a valued nutrient and nutraceutical [1,2]. Omega-3 PUFAs such as EPA are essential in the human diet and important components of lipids, especially in certain organs, such as the central nervous system, playing a beneficial role in the neuronal, retinal, and immune system. They are precursors of resolving eicosanoids and with a well-recognized important role in the regulation of inflammation [3]. This autotrophic microalga accumulates omega-3 PUFAs mainly in polar lipids, such as betaine lipids, glycolipids, and phospholipids [4,5,6,7], which are the main components of cells, thylakoids, and chloroplast membranes. In *N. oceanica,* polar lipids are generally rich in long-chain omega-3 PUFAs (mainly EPA), of high nutritional value, being glycolipids, followed by betaine lipids and phospholipids, which are richer in this omega-3 PUFA than triglycerides [2].

Marine phospholipids offer higher bioaccessibility and bioavailability of omega-3 lipids than triglycerides, and several phospholipids have also been reported with beneficial health effects in the prevention of several chronic diseases, such as cardiovascular diseases [8]. Marine glycolipids from the genus *Nannochloropsis* are also rich in omega-3 lipids and have been associated with anti-tumoral [9,10], antiviral [11,12], antibacterial [13], and anti-inflammatory [14,15,16,17] activities. They have also been described with several beneficial effects on senescence, cognitive functions, and inflammatory diseases, as well as on plasma and hepatic lipid metabolism [18,19]. Marine polar lipids are emerging as new value-added bioactive compounds with high commercial value and applications in the food, functional food and feed, nutraceutical, and pharmaceutical industries [20]. Polar lipids are also good emulsifying agents, making them useful as food products as well as drug excipients widely used in the pharmaceutical and cosmetic industries [21]. In addition, the polar lipids of microalgae, which are the main carriers of omega-3 PUFAs, are also emerging as natural antioxidants. Previous studies have reported the antioxidant potential of polar lipids from microalgae by using both 2,2-diphenyl-1-picrylhydrazyl radical (DPPH^●^) and 2,2′-Azino-bis(3-ethylbenzothiazoline-6-sulfonic acid) (ABTS^●+^) scavenging assays [22,23,24]. Although these methods do not use biologically relevant radicals, they are widely used to screen the antioxidant capacity of potential antioxidant candidates due to their simplicity and the stability of the DPPH^●^ and ABTS^●+^ radicals [25].

To meet the needs of the market, lipid extracts of microalgae are gaining increasing interest due to their unique composition and the possibilities of being directly used as supplements or added to various foods and nutraceuticals, pharmaceuticals, and cosmeceuticals. The nutritional attributes associated with the composition of microalgae may also meet the needs of certain target populations, such as vegans and vegetarians, who require the replacement of ingredients of animal origin with vegetarian ingredients [26]. Many extraction methods have been used to obtain lipid extracts from the biomass of microalgae. Conventional extraction methods use organic solvents to recover total lipids due to their economic and technical advantages; in particular, their high lipid selectivity and solubility, low cost, and relatively easy scaling [27]. Several solvents have been explored in the literature, but a mixture of chloroform and methanol is considered to be the most effective [28]. However, growing awareness of environmental and health risks and legislative restrictions on the presence of chlorinated solvents in food products [29] has led to the search for safe, food-grade, and environmentally safe solvents and extraction procedures. In this context, ethanol is a non-toxic, food-grade, and environmentally friendly solvent that does not pose any regulatory problem in the food industry [30], but whose application in lipid extraction has been little explored due to its low efficiency.

To overcome the limitation of ethanol for lipid extraction, assisted extraction methods have been increasingly explored as a way to improve lipid extraction yields with reduced procedural times and solvent consumption [31]. Among these, ultrasound-assisted extraction emerges as an inexpensive, environmentally friendly, highly efficient, and rapid extraction process that can be scaled to benefit industrial production [30,31]. In our previous work, ultrasonic-assisted ethanol extraction has been shown to be a promising approach, which can be used as an alternative to traditional chlorinated solvents, to obtain EPA-enriched lipid extracts from *N. oceanica* biomass [32].

So far, the impact of extraction methodologies has been primarily studied by fatty acid (FA) composition in total lipid extracts or lipid classes, investigated using low-resolution approaches such as solid-phase extraction (SPE), mainly in the context of biodiesel production [33,34]. However, the effect of ultrasound-assisted extraction on the ability to extract individual polar lipids from microalgae has not been addressed. Thorough knowledge of the molecular composition of lipids is essential to assess the commercial value of environmentally friendly lipid extracts. Such a task can be accomplished using a lipidomics approach based on liquid chromatography–mass spectrometry (LC-MS) [35]. This method has been used successfully to characterize the lipidome profile of various microalgae, including *N. oceanica*, which is the organism studied in this work [4,6,36,37,38].

In this context, the main objective of this study was to evaluate the polar lipid composition at the molecular level of the lipid extracts obtained from *N. oceanica* using different solvent systems and with ethanol extraction assisted by an ultrasonic bath and an ultrasonic probe and to assess the antioxidant properties of the extracts. With this study, we also aimed to improve the knowledge about the composition of ethanolic lipid extracts of *N. oceanica* not only as a source of omega-3 EPA for use in various foods but also as nutritional and pharmaceutical supplements.

## 2. Results

### 2.1. Comparative Analysis of the Polar Lipidome of Extracts of Nannochloropsis oceanica Obtained Using Different Solvent Systems

In this study, we determined the polar lipid profile of lipid extracts obtained from *N. oceanica* biomass using different lipid solvent extraction methods (chloroform/methanol (CM), dichloromethane/methanol (DM), dichloromethane/ethanol (DE), and ethanol (E)) and ultrasound-assisted methodologies (ethanol extraction assisted with bath (E + USB) or probe (E + USP)). An additional extraction step based on the Folch’s method was performed before the HPLC and the activity experiments to remove small amounts of other non-lipid components (such as peptides and carbohydrates). This step made it possible to accurately assess the lipid content of the ethanolic extracts without altering its lipid composition.

A total of 128 lipid species were identified in *N. oceanica* lipid extracts by retention time, mass accuracy, and MS/MS data (Supplementary Materials, Table S1), including 15 monogalactosyl diacylglycerol (MGDG), 5 monogalactosyl monoacylglycerol (MGMG), 14 digalactosyl diacylglycerol (DGDG), 2 digalactosyl monoacylglycerol (DGMG), 10 sulfoquinovosyl diacylglycerol (SQDG), 1 sulfoquinovosyl monoacylglycerol (SQMG), 19 diacylglyceryl-N,N,N-trimethyl homoserine (DGTS), 4 monoacylglyceryl-N,N,N-trimethyl homoserine (MGTS), 11 phosphatidylglycerol (PG), 6 phosphatidylinositol (PI), 10 phosphatidylethanolamine (PE), 20 phosphatidylcholine (PC), 9 lyso phosphatidylcholine (LPC), and 2 phosphoinositol ceramide (PI-Cer) species. All species were identified in all extracts and were used to assess the lipid extraction efficiency of the different methods. The polar lipid species identified in this study have all been previously reported in the literature for *Nannochloropsis* species, including *N. oceanica* [6,36,37,38,39,40], except for the DGMG(16:1), DGMG(16:0), LPC(14:0), LPC(16:2), LPC(18:3), and PC(36:7) lipid species, which are reported here for the first time.

**Figure 1:**
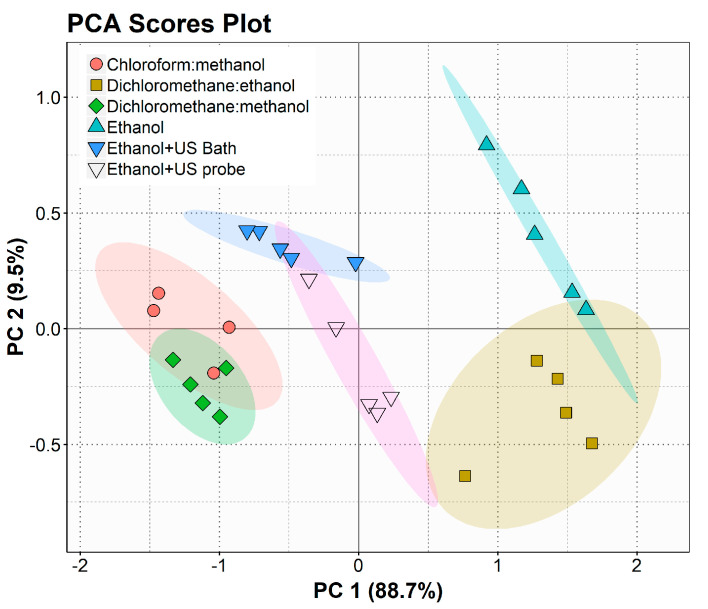
Principal component analysis (PCA) score plot of the polar lipid classes identified by HILIC-LC-Q-Exactive-MS in the lipid extracts obtained with different solvent systems: chloroform/methanol (DM), dichloromethane/ethanol (DE), dichloromethane/methanol (DM), and ethanol (E); ultrasonic-assisted methodologies (US): ethanol + US Bath and ethanol + US probe.

For the lipid class, principal component analysis (PCA) score plots were constructed (Figure 1) to depict the variability in polar lipid profiles on the different extracts, as shown in Supplementary Materials, Table S2. The projection of the resulting sample scores for the first and second principal components together accounted for 98.2% of the total sample variance. The separation was mainly due to PC1, which accounted for 88.7% of the data variability. PCA showed that the CM and DM extracts were closely clustered on the left side, highlighting their similar composition. Extracts E and DE are present on the right side of the plot, clearly apart from the two conventional methods and almost separated from each other. Ultrasound-assisted ethanol extracts can be found in the central area of the plot but deviated to the left side of the plot. These results reveal greater similarity between the ethanolic ultrasound-assisted extracts and the conventional chlorinated solvent extracts than the dichloromethane/ethanol and ethanol extracts.

**Figure 2:**
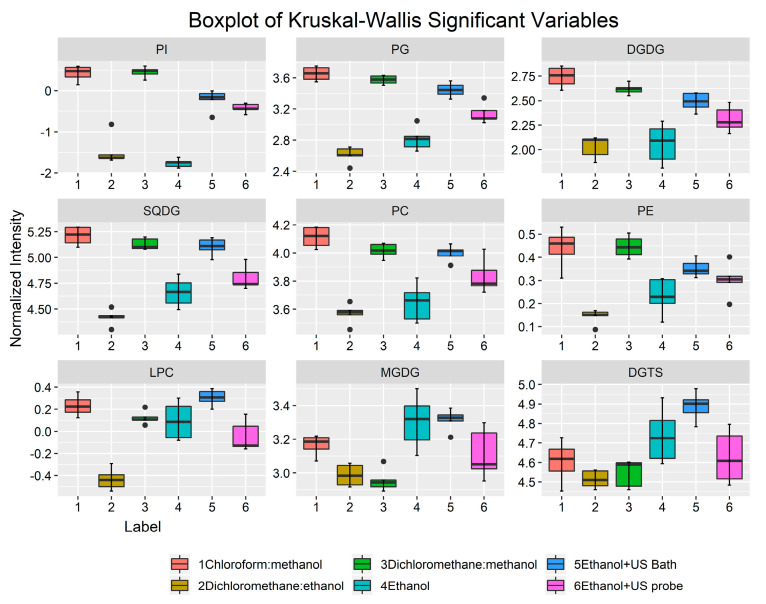
Boxplot of the polar lipid classes identified in lipid extracts of *Nannochloropsis oceanica* obtained using different lipid solvent extraction methods and ultrasound-assisted methodologies. The thick bar is the median value, and dots represent the outliers of five replicates. PC—phosphatidylcholine; LPC—lysophosphatidylcholine; PE—phosphatidylethanolamine; PI—phosphatidylinositol; PG—phosphatidylglycerol; DGDG—digalactosyl diacylglycerol; MGDG—monogalactosyl diacylglycerol; SQDG—sulfoquinovosyl diacylglycerol; DGTS—diacylglycerol-N,N,N-trimethyl homoserine.

A univariate statistical analysis was then performed to assess the lipid class that most contributes to the discrimination of the extracts (Supplementary Materials, Tables S3 and S4). Figure 2 shows that the lipid classes PI, PG, DGDG, and SQDG contribute the most to the discrimination of the extracts. For these classes, it is possible to observe the great discrimination between the extracts obtained with the chlorinated solvents, CM and DM, and the extracts of DE and E. On the other hand, the ultrasound-assisted extracts, in particular the extract obtained with ethanol assisted by ultrasound bath, show increasing similarity to their chlorinated analogues.

**Figure 3:**
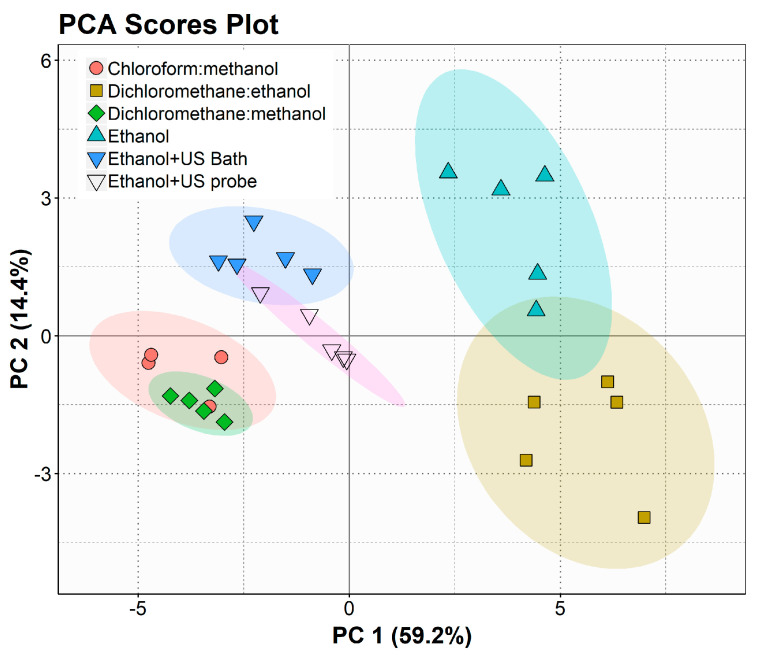
Principal component analysis (PCA) score plot of the polar lipid species identified by HILIC-LC-Q-Exactive-MS in lipid extracts produced with different solvent systems: chloroform/methanol (DM), dichloromethane/ethanol (DE), dichloromethane/methanol (DM), ethanol (E), ultrasonic bath assisted methodologies (ethanol + US Bath), and ultrasonic probe assisted methodologies (ethanol + US probe).

For a more detailed interpretation of the data, statistical analysis was also performed using the relative abundances of lipid species in the different extracts, shown in Supplementary Materials, Table S5. As observed in Figure 3, the PCA plot also shows the clustering of the CM and DM extracts and the E and DE extracts on opposite sides of the plot. However, it is possible to observe a tighter cluster of the extracts obtained using ultrasound and a greater proximity to the cluster of chlorinated solvents.

**Figure 4:**
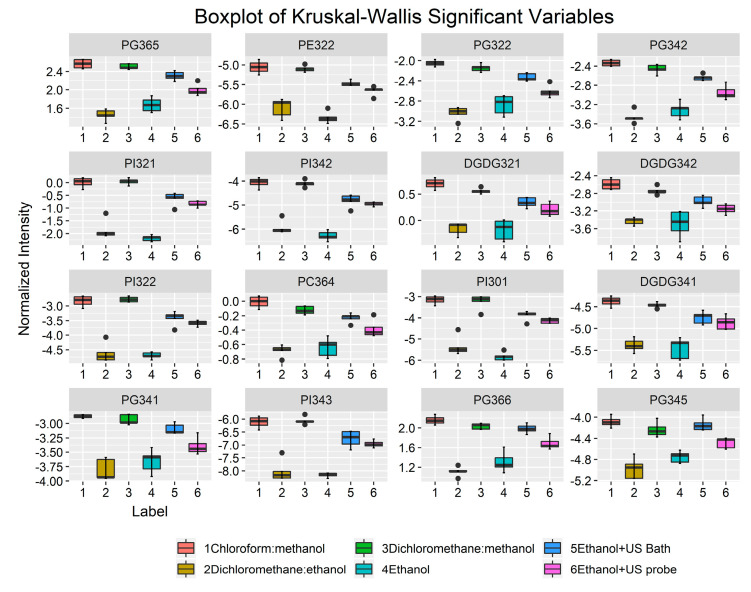
Boxplot of the 16 most discriminating polar lipid species for PC1 from lipid extracts of *Nannochloropsis oceanica* obtained using different solvent systems and ultrasound-assisted methodologies. For all lipid species q < 0.05. The thick bar is the median value, and the dots are the outliers of five replicates. PC—phosphatidylcholine; PE—phosphatidylethanolamine; PI—phosphatidylinositol; PG—phosphatidylglycerol; DGDG—digalactosyl diacylglycerol.

Univariate statistical analysis showed that 16 individual lipid species, including 6 PG, 5 PI, 3 DGDG, 1 PE, and 1 PC, contributed the most to the discrimination of the extracts (Figure 4 and Supplementary Material, Table S6). These results corroborate those obtained for the lipid classes, in which the statistical analysis also attributed the greatest variability among the extracts mainly to the lipid classes PI, PG, and DGDG. In addition, three of the most discriminating species whose concentrations in the extracts obtained with ethanol ultrasonic-assisted extraction are closer to those obtained with conventional methods, namely PG 34:5 (PG 14:0_20:5), PG 36:5 (PG 16:0_20:5), and PG36:6 (PG 16:1_20:5), contain omega-3 fatty acid EPA in their composition. A similar trend is observed for all discriminant species, according to which there are no statistical differences between the ultrasound-assisted ethanolic extracts and their chlorinated analogues (q > 0.05).

### 2.2. Effect of Different Extraction Methodologies on the Antioxidant Profile of the Polar Lipid Extracts of Nannochloropsis oceanica

**Figure 5:**
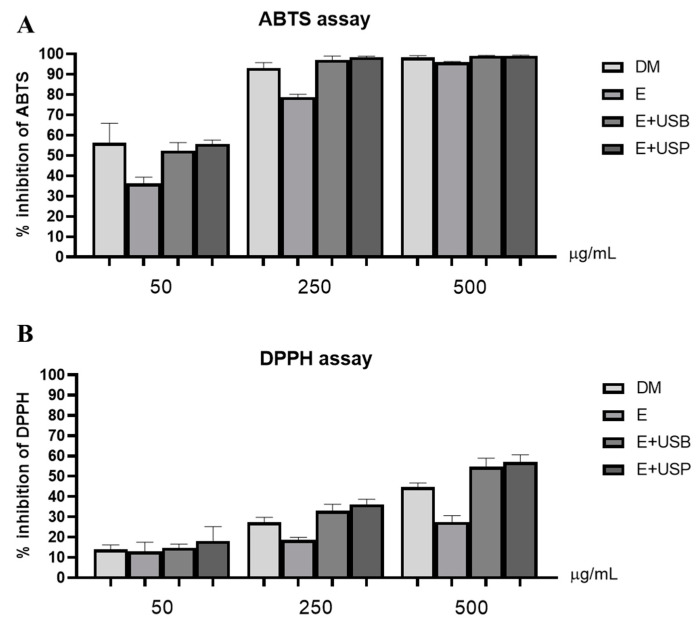
Percentage inhibition of ABTS^●+^ (**A**) and DPPH^●^ (**B**) obtained in the presence of polar lipid extracts of *Nannochloropsis oceanica* extracted with dichloromethane/methanol (DM), ethanol (E), ethanol assisted by an ultrasonic bath (E + USB), and ethanol assisted by an ultrasonic probe (E + USP) (50, 250, and 500 μg/mL in ethanol) after 120 min of reaction. Values are presented as the average of three assays (*n* = 3) ± standard deviation.

The antioxidant potential of the polar lipid extracts of *N. oceanica* extracted using dichloromethane/methanol (DM), ethanol (E), ethanol assisted with ultrasonic bath (E + USB), and ethanol assisted with ultrasonic probe (E + USP) were screened using the ABTS and DPPH radical scavenging assays (Figure 5).

The scavenging capacity depended on the concentrations studied (50, 250, and 500 μg/mL), with a non-linear antioxidant response. In both assays (ABTS and DPPH), the polar lipid extracts obtained with ethanol were less effective as scavengers. Otherwise, the polar lipid extracts obtained using E + USP and E + USB showed an antioxidant profile similar to that obtained using conventional extraction methodology with DM. This highlights the ability of ethanol ultrasonic-assisted extraction as a potential source of value-added polar lipids.

## 3. Discussion

This work showed the comparison of the polar lipid profile in lipid extracts obtained from the biomass of *N. oceanica* with different solvent systems: chloroform/methanol (CM), dichloromethane/methanol (DM), dichloromethane/ethanol (DE), and ethanol (E), as well as ethanol extraction assisted by an ultrasonic bath (E + USB) and an ultrasonic probe (E + USP). The main objective was to provide a comprehensive overview of lipid extraction using different solvent systems and to investigate the use of ethanol, an environmentally friendly and food-grade solvent, as a means of obtaining omega-3 EPA (Supplementary Materials, Table S1) and bioactive lipids for distinct biotechnological applications.

CM extraction is the conventional approach used to obtain extracts of polar lipids from microalgae used in different methods, such as Bligh and Dyer and Folch extraction [41]. Chloroform is known for its safety concerns resulting from its recognized toxicity and its harmful effects on the environment [42]. These challenges with this conventional solvent prompted the use of dichloromethane as a substitute of lower toxicity, even in research studies where no limitation of solvents is imposed, to overcome the drawbacks of using chloroform without compromising the extraction efficiency [43,44]. Indeed, the CM extract, used in our study as a reference, showed a polar lipid profile comparable to the DM extract (q < 0.05) (Figure 1, Supplementary Materials, Table S4) and a richness in EPA lipids (Supplementary Materials, Table S1). Comparable results have been described by Stranska-Zachariasova and co-authors for the microalga *Trachydiscus minutus* [45]. However, extracts obtained with chlorinated solvents, such as chloroform or dichloromethane, are not suitable for food purposes and for large-scale applications in the nutraceutical and pharmaceutical industries. Thus, there is a need for sustainable, green, and environmentally friendly options for these applications.

Ethanol is recognized as a food-grade, environmentally friendly, and safe solvent, and therefore widely recommended to meet food and nutraceutical applications of lipid extracts [28]. However, ethanol has already been shown to have lower efficiency in lipid extraction as well as in EPA recovery efficiency [32,46]. In this study, we also observed the overall poor performance of ethanol in the extraction of polar lipids (Figure 2). To overcome this and improve the extraction yields, the extracts were prepared using extraction with ethanol assisted by an ultrasonic bath (E + USB) and a probe (E + USP). Indeed, it has been reported that the use of ultrasonic-assisted solvent extraction increases the amount of lipids extracted from microalgal biomass as it can facilitate cell membrane disruption and increase the access of solvents to lipids [31]. This made it possible to obtain a polar lipid profile very similar to that of the conventional methods, namely, the CM and DM extracts (q < 0.05) (Supplementary Materials, Table S4). Statistical analysis demonstrated that PI, PG, DGDG, and SQDG were the lipid classes that contributed the most to the discrimination of the different lipid extracts produced using different extraction procedures (Supplementary Materials, Table S3), with the ultrasound-assisted extracts being closer to conventional methodologies (Figure 1). These classes of polar lipids were in higher amounts when ethanol combined with USP and USB was used compared to ethanol, reaching amounts similar to those of DM and CM, and thus showing that the ethanol-assisted extraction is an alternative method to conventional methods. The lipid species of these classes are carriers of omega-3 PUFAs. Analysis of the polar lipid profile revealed that several molecular species of phospholipid contained the omega-3 EPA, with particular emphasis on lipid molecular species belonging to the PG class (Supplementary Materials, Table S1), which were also more extracted with ethanol-assisted extraction than with ethanol (Figure 4). For example, we observed an enrichment of the E + USB and E + USP extracts in PG molecular species containing the omega-3 EPAs, namely, PG(36:5), PG(36:6), and PG(34:5), by comparison with the extracts obtained with ethanol (Figure 4). Overall, the use of ultrasound has made it possible to recover the valuable polar lipids with omega-3, namely, EPA.

The results collected with the fingerprinting analysis of the polar lipidome of *N. oceanica* obtained using different extraction methodologies demonstrated the similarity between the extracts obtained with chloroform/methanol and dichloromethane/methanol. Moreover, compared to ethanol extraction, ethanol-assisted extraction shifted the polar lipid profile towards a greater resemblance to conventional extraction methods (Figure 1 and Figure 3), being a suitable alternative to obtain food-grade and environmentally friendly polar lipid extracts rich in valuable lipids for distinct high-end applications.

Polar lipids, including phospholipids and glycolipids, are emerging as sources of new value-added bioactive compounds with recognized nutritional value and health benefits. The use of phospholipids as value-added ingredients for food fortification is increasingly well known due to its many benefits. Phospholipids of marine origin, especially algae, are effective carriers of omega-3 FA (such as EPA), increasing the bioavailability of these essential lipids compared to other dietary forms of omega-3 PUFAs [8]. It is well documented that EPA is an essential omega-3 PUFA for human nutrition and health [47]. As humans are unable to synthesize these PUFAs, it is crucial that these omega-3 lipids are provided through food and food supplements. This will aid in the nutrient supply of these omega-3 PUFAs, contributing to increasing the bioactivity of EPA-rich lipids such as the prevention of anti-inflammatory and cardiovascular diseases and the maintenance of healthy cognitive functions [18,47,48]. The EPA-rich polar lipid species that have been identified here in the polar lipidome of *N. oceanica* have also been reported in the literature to play an important role in several biological activities. The PG(36:5) species were found to possess anti-inflammatory properties assessed by a strong inhibitory activity of nitric oxide (NO) against the production of NO, induced by lipopolysaccharide in macrophage cells RAW264.7 [49]. In addition, the application of phospholipids is well recognized in the cosmetic and nutraceutical industries, where they are used as key ingredients in emulsifiers, liposome formers, solubilizers, and wetting agents [50,51].

Glycolipids from microalgae are also a reservoir of nutritional omega-3 PUFAs and bioactive compounds with a variety of reported health benefits and bioactivities [52]. The DGDG species, namely, DGDG (20:5/14:0), DGDG (20:5/16:0), DGDG (20:5/16:1), and DGDG (20:5/20:5), and their monogalactosyl (MGDG) analogues, isolated from the glycolipid extract of *Nannochloropsis granulata*, demonstrated strong anti-inflammatory activity [14].

The betaine lipids DGTS (40:10), DGTS (40:9), DGTS (38:7), DGTS (36:6), DGTS (36:5), and DGTS (34:5) from *Nannochloropsis granulata*, also identified in the ethanol US extracts of *N. oceanica*, have also been reported with anti-inflammatory properties [53]. Lyso-diacylglyceryltrimethylhomoserine MGTS (20:5) has been identified as the compound responsible for increasing the activity of Paraoxonase 1 (PON1) in macrophages, an enzyme known to exert beneficial effects in several processes related to atherosclerosis thanks to its strong anti-inflammatory and antioxidant activities [54].

Although the raw biomass of *N. oceanica* is still not approved for human consumption, it is rich in beneficial lipids and bioactive compounds. Therefore, its extracts are a promising source of functional ingredients for the food, nutraceutical, and pharmaceutical industries. We found that the food-grade and environmentally friendly ethanol extracts (E + USB, and E + USP) required for these uses resulted in a polar lipid signature similar to conventional extraction methods. These extracts have been shown to be a potential source of value-added polar lipids with antioxidant properties. These results demonstrated the potential of these polar lipid extracts to be exploited also as a source of bioactive compounds. Therefore, polar lipid extracts are also a promising source of compounds with high potential for biotechnological exploitation. However, it is also important to note that we cannot rule out the presence of other compounds with recognized antioxidant properties, such as polyphenols and pigments, in these extracts, which may synergistically contribute to the overall activity observed. In addition, the additional extraction step based on the Folch extraction performed in this study did not change the lipid composition obtained with E + USP and E + USB. Nevertheless, it is important to stress that this procedure, which is not to be used in an industrial context because it uses non-food-grade extraction solvents, can impact to a certain extent the antioxidant potential demonstrated by the extracts, measured in this work. Prior to extraction by the Folch method, other non-lipid components may be present in the extracts, which may impair or contribute to the overall antioxidant activity.

Overall, our results have shown that ultrasonic-assisted ethanol extraction may be an appropriate way to obtain food-grade and green polar lipid species from the biomass of *N. oceanica*, which is rich in omega-3 EPA, for applications as functional food ingredients or in nutraceuticals and pharmaceuticals. Ethanol-assisted extraction (E + USB and E + USP) has proven to be an effective alternative to conventional lipid extraction methodologies, providing polar lipid extracts rich in EPA with a composition similar to that of CM and DM extractions. Therefore, ultrasound-assisted ethanol extraction can be seen as a promising method to obtain food-grade polar lipids for high-end biotechnology applications, with the benefit of the high potential of ultrasound-assisted extraction to be scaled-up at the industrial level [55]. These lipid extracts, which are very rich in EPA, can be used as food ingredients or as ingredients of food supplements and nutraceuticals, to fortify these products with EPA. The consumption of foods enriched in EPA contributes to a healthier diet and can be used as a strategy for the prevention of diseases through diet [56]. On the other hand, the presence of certain lipid species that have been associated with pharmacological activities, such as anti-inflammatory properties (e.g., PG (16:0/20:5), DGDG (20:5/14:0), DGDG (20:5/16:0), DGDG (20:5/16:1), and DGDG (20:5/20:5)), open new paths for the investigation of new bioactive compounds and drugs for future application in cosmeceuticals and pharmaceutical industries. However, this remains a poorly explored area of research and much more isolation, characterization, and bioprospecting studies are needed to isolate and identify the bioactive molecules in order to unveil the true structure–activity relationship and design new applications.

## 4. Materials and Methods

### 4.1. Algal Material

Spray-dried biomass of *Nannochloropsis oceanica* was supplied by Allmicroalgae, Natural Products S.A., located in Pataias, Portugal. *Nannochloropsis oceanica* was grown in Guillard’s F/2 medium at 0.31 g/L of NO_3_ˉ supplemented with 12 µM of iron, 30 g/L of NaCl (Salexpor, Coimbra, Portugal), and magnesium (Necton, Faro, Portugal) [57]. All the microalga biomass used in this study was obtained from the same cultivation batch.

### 4.2. Reagents

HPLC-grade chloroform (CHCl_3_), methanol (MeOH), dichloromethane (CH_2_Cl_2_), and ethanol (EtOH, 96%) were purchased from Fisher Scientific Ltd. (Loughborough, UK). All other reagents were purchased from major commercial sources and used without further purification. (±) 6-hydroxy-2,5,7,8-tetramethylchromane-2-carboxylic acid (Trolox) was purchased from Sigma-Aldrich (St Louis, MO, USA). 2,2-diphenyl-1-picrylhydrazy radical (DPPH^●^) was purchased from Aldrich (Milwaukee, WI). 2,2′-Azino-bis(3-ethylbenzothiazoline-6-sulfonic acid) diammonium salt (ABTS^●+^) was obtained from Fluka (Buchs, Switzerland). 1,2-dimyristoyl-*sn*-glycero-3-phosphocholine (dMPC), 1,2-dimyristoyl-*sn*-glycero-3-phosphoethanolamine (dMPE), N-heptadecanoyl-D-*erythro*-sphingosyl-phosphorylcholine (SM d18:1/17:0), 1-nonadecanoyl-2-hydroxy-*sn*-glycero-3-phosphocholine (LPC), 1,2-dipalmitoyl-*sn*-glycero-3-phosphatidylinositol (dPPI), 1,2-dimyristoyl-*sn*-glycero-3-phospho-(10-rac-) glycerol (dMPG), 1,2-dimyristoyl-*sn*-glycero-3-phospho-L-serine (dMPS), 1,2-dimyristoyl-*sn*-glycero-3-phosphate (dMPA), and C17 Ceramide (d35:1) were purchased from Avanti Polar Lipids, Inc. (Alabaster, AL). Milli-Q water (Synergy, Millipore Corporation, Billerica, MA, USA) was used.

### 4.3. Lipid Extraction Procedure

Lipid extraction was performed using chloroform/methanol (CM) (2:1, *v/v*), dichloromethane/methanol (DM) (2:1, *v/v*), and dichloromethane/ethanol (DE) (2:1, *v/v*) or ethanol (E).

Briefly, a volume of 1 mL of each of the solvent mixtures (CM, DM, or DE) or E was added to an amount of 50 mg of *N. oceanica* biomass. The samples were then incubated at 30 °C for 30 min in a heather block (Stuart^®^ SBH200D/3, Bibby Scientific, Ltd., Stone, Staffordshire, ST15 OSA, UK). After the incubation time, the suspension was centrifuged (Selecta JP Mixtasel, Abrera, Barcelona, Spain) at 626× *g* for 10 min and the supernatant was collected in a new pre-weighed glass tube. This process was repeated 8 times with the organic phases all combined in the same pre-weighted tube. The combined organic phases (total extract) were dried under a stream of N_2_ and the tubes were weighted.

To remove the non-lipid contaminants, the total extracts were subjected to a purification process based on the Folch extraction [28]. Therefore, the total extracts were re-dissolved in 2 mL of chloroform and 1 mL of methanol and 0.75 mL of Milli-Q water were added. The mixture was vortexed for 1 min followed by phase separation by centrifugation (Selecta JP Mixtasel, Abrera, Barcelona, Spain) at 626× *g* for 10 min. The organic phase was collected in a new pre-weighed tube and the aqueous phase was re-extracted with 2 mL of chloroform. The combined organic phases (lipid extract) were dried under a stream of N_2_ and weighted.

Each series of samples was repeated five times. In all the approaches, the total content of the extracts obtained from the dry biomass as well as the content of purified lipid extracts were determined by gravimetry.

### 4.4. Ultrasound-Assisted Ethanol Total Lipid Extraction

In an attempt to increase the extraction efficiency of ethanol, an ultrasound bath and ultrasound probe were used. Each set of samples was repeated five times and the total lipid content was gravimetrically determined.

#### 4.4.1. Ultrasound Bath-Assisted Extraction

An amount of 50 mg of dried biomass was combined with 1 mL of ethanol in a 15 mL Pyrex glass tube. Samples were placed in an ultrasonic water/ice bath working at 50 kHz (J. P Selecta S.A. Model 3000683, 2.6 L, internal dimensions: 9 × 23 × 13 cm) for 30 min, with periodical temperature control in order not to exceed 30 °C. After this time, samples were centrifuged at 626× *g* for 10 min to pellet the residual biomass. The organic phase was collected to a new pre-weighed tube, dried under N_2_ stream, and weighed. This process was repeated 8 times with the organic phases all combined into the same pre-weighted tube, dried under N_2_ stream, and weighed. To eliminate non-lipid contaminants, the extracts were subjected to a purification process based on Folch extraction [28], as described above.

#### 4.4.2. Ultrasound Probe-Assisted Extraction

An amount of 50 mg of dried biomass was combined with 1 mL of ethanol in a 15 mL test tube. The samples were sonicated with a Sonics VCX 130 sonifier equipped with a microtip probe set to 70% amplitude for six 20 s pulses, with each pulse followed by a 1 min cooldown period in ice water. Total sonication time was 8 min. This process was repeated 8 times. Samples were then centrifuged at 626× *g* for 10 min to pellet the residual biomass. The organic phase was collected to a new pre-weighed tube, dried under N_2_ stream, and weighed. To eliminate non-lipid contaminants, the extracts were subjected to a purification process based on Folch extraction [28], as described above.

### 4.5. Polar Lipid Analysis by HILIC-LC-Q-Exactive-MS

Lipid extracts were separated using a high-performance liquid chromatography (HPLC) system with an autosampler (Ultimate 3000 Dionex, Thermo Fisher Scientific, Bremen, Germany) coupled online to a Q-Exactive^®^ hybrid quadrupole Orbitrap^®^ mass spectrometer (Thermo Fisher Scientific, Bremen, Germany), according to [58,59].

The solvent system consisted of two mobile phases: mobile phase A (acetonitrile:methanol:water 50:25:25 (*v/v/v*) with 1 mM ammonium acetate) and mobile phase B (acetonitrile:methanol 60:40 (*v/v*) with 1 mM ammonium acetate). The gradient started with 40% of mobile phase A, which was held isocratically for 8 min. Then, a linear increase to 60% of A occurred within 7 min followed by a maintenance period of 5 min. The returning to the initial conditions occurred in 5 min, followed by a re-equilibration period of 10 min prior next injection.

A volume of 5 µL of each sample, containing 5 µg of lipid extract, 4 µL of internal standard mix (dMPC—0.02 µg, dMPE—0.02 µg, SM d18:1/17:0—0.02 µg, LPC—0.02 µg, dPPI—0.08 µg, dMPG—0.012 µg, dMPS—0.04 µg, dMPA—0.08 µg, and C17 Ceramide (d35:1) —0.02 µg), and 91 µL of solvent system (60% of eluent B and 40% of eluent A) was introduced into the Ascentis^®^ Si column (15 cm × 1 mm, 3 µm, Sigma-Aldrich) with a flow rate of 40 µL min^−1^ and at 30 °C.

The mass spectrometer with Orbitrap^®^ technology operated simultaneously in positive (electrospray voltage 3.0 kV) and negative (electrospray voltage −2.7 kV) modes with a high resolution of 70,000 and AGC target of 1 × 10^6^. The capillary temperature was 250 °C and the sheath gas flow was 15 U. In the MS/MS experiments, a resolution of 17,500 and AGC target of 1 × 10^5^ were used, and the cycles consisted of one full scan mass spectrum and ten data-dependent MS/MS scans that were repeated continuously throughout the experiments, with the dynamic exclusion of 60 s and intensity threshold of 1 × 10^4^. Normalized collision energy™ ranged between 25, 30 and 35 eV. All analyses were performed five times. At least one blank run was performed between different extract samples to prevent cross-contamination. Data acquisition was carried out using the Xcalibur data system (V3.3, Thermo Fisher Scientific, Waltham, MA, USA).

### 4.6. Data Analysis

LC-MS data analysis was performed with MZmine v2.32 software [60] using the parameters previously reported [58]. The raw LC-MS data were processed by filtering and smoothing, peak detection, peak processing, and assignment against an in-house lipid data bank, created using the LIPID MAPS database [61]. All the chromatographic peaks with a raw intensity lower than 1 × 10^4^ were excluded. For all assignments, the typical retention time of the respective lipid class and accurate mass measurements (peaks within 5 ppm of the lipid exact mass) were considered. The monogalactosyl diacylglyceride (MGDG) and digalactosyl diacylglyceride (DGDG) species, and their lyso forms monogalactosyl monoacylglyceride (MGMG) and digalactosyl monoacylglyceride (DGMG), respectively, were analyzed in the LC-MS spectra in the positive mode as ammonium adducts, [M + NH_4_]^+^ ions. Monoacylglyceryl-N,N,N-trimethyl homoserine (MGTS), diacylglyceryl-N,N,N-trimethyl homoserine (DGTS), phosphatidylcholine (PC), lysophosphatidylcholine (LPC), and phosphatidylethanolamine (PE) species were also analyzed in the LC-MS spectra in the positive mode as [M + H]^+^ ions. Sulfoquinovosyldiacylglyceride (SQDG), sulfoquinovosylmonoacylglyceride (SQMG), phosphatidylglycerol (PG), phosphatidylinositol (PI), and phosphoinositol ceramide (PI-Cer) species were analyzed in the LC-MS spectra in the negative ion mode, as [M − H]^−^ ions. For normalization of the LC-MS data, the values of the integrated peak areas of the extracted ion chromatograms (XIC) of each polar lipid species was divided by the value of the peak area of the lipid internal standard with the closest retention time and selected for each lipid class.

The validation of the assignments and the confirmation of the molecular composition of the distinct lipid species was performed based on the analysis of the LC-MS/MS data acquired both in positive and negative ion mode, as previously described [62]. The LC-MS/MS data of the [M + H]^+^ ions of PC, LPC, and PE allowed confirming the polar head groups of these lipid classes. The fatty acyl composition was confirmed by analysis of the LC-MS/MS spectra of the acetate adducts, [M + CH_3_COO]^−^ ions, for the PC and LPC species, and [M − H]^−^ ions for the PE species, which allowed the identification of the carboxylate anions (RCOO^−^) of the fatty acyl chains. The tandem mass spectra of the [M + H]^+^ ions of DGTS and lyso DGTS (MGTS) as well as of the [M + NH_4_]^+^ ions of MGDG, MGMG, DGDG, and DGMG made it possible to confirm the identity of the polar head group and the fatty acyl chain(s) of the molecular species belonging to these lipid classes. For DGTS and MGTS, fatty acyl chains were identified due to their typical neutral losses as an acid (RCOOH) or ketene (R=C=O) derivative while for MGDG, MGMG, DGDG, and DGMG, the fatty acyl chains were identified due to the presence of acylium ions plus 74 Da [RCO + 74]^+^. The analysis of the LC tandem mass spectra of the [M − H]^−^ ions of the lipid species belonging to SQDG and its lyso form SQMG, PI, and PG classes allowed the confirmation of the polar head groups and of the respective fatty acyl chain(s), which were assigned by the identification of their carboxylate anions (RCOO^−^). PI-Cer were also analyzed as [M − H]^−^ ions [63]. The attribution of the position *sn-*1 and *sn-*2 was performed according to the LIPID MAPS classification, where “/” indicates that the *sn-*position of the fatty acyl chains is known while “_” indicates that the *sn-*position is not known [64], and it was assigned taking into account the main biosynthetic pathways of each class of polar lipids.

### 4.7. ABTS^●+^ Scavenging Activity

The microplate ABTS^●+^ scavenging assay was performed as previously described by Melo and co-authors [65]. The ABTS^●+^ (3.5 mmol/L) was prepared by mixing 10 mL of ABTS stock solution (7 mmol/L in Milli-Q water) with 10 mL of potassium persulfate (2.45 mmol/L in Milli-Q water). This solution was kept in the dark, at room temperature, for 16 h. Before analysis, the ABTS^●+^ (3.5 mmol/L) was diluted in ethanol to obtain a diluted solution with an absorbance of 0.9 at 734 nm, measured using a UV–vis spectrophotometer (Multiskan GO 1.00.38, Thermo Scientific, Hudson, NH, USA) controlled by the SkanIT software version 3.2 (Thermo Scientific). The stability of the diluted ABTS^●+^ solution was assessed by adding a volume of 150 µL of ethanol and 150 µL of ABTS^●+^ to 16 microplate wells and measuring the absorbance at 734 nm every 5 min during 120 min. The ABTS^●+^ solution with an absorbance decline of less than 10% were considered stable. To evaluate the radical scavenging ability, 150 µL of polar lipid extracts from *N. oceanica* were extracted using dichloromethane/methanol (DM), ethanol (E), ethanol assisted with bath (E + USB), and ethanol assisted with probe (E + USP) (100, 500, and 1000 μg/mL in ethanol), or 150  μL of Trolox standard solutions (between 5 and 75  μmol/L in ethanol) were placed in each well and mixed with 150 µL of ABTS^●+^. Control samples were also performed by replacing the volume of ABTS^●+^ with ethanol. Samples were incubated for 120 min, with measurement of the absorbance at 734 nm every 5 min. All measurements were performed in triplicate.

The amount of ABTS^●+^ (%) remaining was calculated as follows:

% ABTS^●+^ remaining = (Abs samples after incubation time/Abs sample at the beginning of reaction) × 100

The free radical-scavenging activity was determined as the percentage of inhibition of ABTS^●+^:

% Inhibition = ((Abs ABTS^●+^ – (Abs samples – Abs control samples))/Abs ABTS^●+^) × 100

### 4.8. DPPH^●^ Scavenging Activity

The microplate DPPH^●^ scavenging assay was performed as previously described by Melo and co-authors [65]. The stock solution of DPPH^●^ (250 mmol/L) was prepared in ethanol. This solution was diluted in ethanol to obtain a diluted solution with an absorbance of 0.9 at 517 nm, measured using a UV–vis spectrophotometer (Multiskan GO 1.00.38, Thermo Scientific, Hudson, NH, USA) controlled by the SkanIT software version 3.2 (Thermo Scientific). The stability of the diluted DPPH^●^ solution was assessed by adding a volume of 150 µL of ethanol and 150 µL of DPPH^●^ to 16 microplate wells and measuring the absorbance at 517 nm every 5 min during 120 min. The DPPH^●^ solution with an absorbance decline of less than 10% was considered stable. To evaluate the radical scavenging ability, 150 µL of polar lipid extracts from *N. oceanica* were extracted using dichloromethane/methanol (DM), ethanol (E), ethanol assisted with bath (E + USB), and ethanol assisted with probe (E + USP) (100, 500, and 1000 μg/mL in ethanol), or 150  μL of Trolox standard solutions (between 5 and 75  μmol/L in ethanol) were placed in each well and mixed with 150 µL of DPPH^●^. Control samples were also performed by replacing the volume of DPPH^●^ with ethanol. Samples were incubated for 120 min, with measurement of the absorbance at 517 nm every 5 min. All measurements were performed in triplicate.

The amount of DPPH^●^ (%) remaining was calculated as follows:

% DPPH^●^ remaining = (Abs samples after incubation time /Abs sample at the beginning of reaction) × 100

The free radical-scavenging activity was determined as the percentage of inhibition of DPPH^●^:

% Inhibition = ((Abs DPPH^●^– (Abs samples – Abs control samples))/Abs DPPH^●^) × 100

### 4.9. Statistical Analysis

Statistical analyses, including the multivariate and univariate analyses, were performed using R version 4.1.0 [66] in Rstudio version 1.4 [67]. LC-MS data were glog transformed, EigenMS normalized [68], and autoscaled using the R package MetaboAnalyst [69]. Principal component analysis (PCA) was performed with the R package pcaMethods [70]. Kruskal–Wallis tests were used for nonparametric comparisons of median values among the six groups, followed by a nonparametric pairwise multiple-comparison procedure using the Dunn test. These tests were performed with the R built-in functions. *p*-values were corrected for multiple testing using the BH Benjamini, Hochberg, and Yekutieli method (q values) [71]. PCA and boxplot graphics were created using the R package ggplot2 [72].

## 5. Conclusions

The present results demonstrated the impact of different extraction solvents in the polar lipid composition of the resulting extracts. The E + USB and E + USP extracts were unveiled as promising, efficient, green, and food-grade alternatives to conventional CM and DM extracts for obtaining high-value-added extracts for applications in the pharmaceutical, nutraceutical, and functional food industries. In addition, the identification of polar lipid molecules with high added value contributes to increasing the knowledge of lipid extracts from the microalga *N. oceanica* as an important source of bioactive agents for biotechnological applications.

## Data Availability

The data presented in this study are available on request from the corresponding authors.

## Supplementary Materials

The following are available online at https://www.mdpi.com/article/10.3390/md19110593/s1, Table S1: Polar lipid species identified in the lipidome of *Nannochloropsis oceanica* by HILIC–MS and MS/MS, with information on the type of ion, lipid identity, calculated mass, observed mass, mass error (Δ ppm), fatty acyl composition, and molecular formula. The glycolipid species belonging to MGDG, MGMG, DGDG, and DGMG classes were identified as [M + NH_4_]^+^ ions; SQDG and SQMG were identified as [M − H]^−^ ions. Phospholipid species of PC, LPC, and PE as well as betaine lipids from DGTS and MGTS classes were identified as [M + H]^+^ ions while the lipid species from phospholipid classes PG and PI and sphingolipids PI-Cer were identified as [M − H]^−^; ions. In the fatty acyl chains, C represents the total number of carbon atoms and N represents the total number of double bonds. The C:N/C:N assignment indicates the attribution of fatty acyl chains to the position *sn*-1/*sn*-2, while in C:N_C:N assignment the attribution of the *sn*-1 and *sn*-2 position of fatty acyl chains is not known. * Assignments by mass accuracy, unequivocal molecular formula, and typical retention time of lipid class, but without MS/MS, Table S2. Main contributors for the PCA first dimension allowing discrimination between the groups based on polar lipid classes. Abbreviations: monogalactosyl diacylglycerol–MGDG, digalactosyl diacylglycerol–DGDG, sulfoquinovosyl diacylglycerol–SQDG, phosphatidylcholine–PC, lyso phosphatidylcholine–LPC, phosphatidylethanolamine–PE, phosphatidylglycerol–PG, phosphatidylinositol–PI, diacylglyceryl-O-4′-(N,N,N-trimethyl) homoserine–DGTS, Table S3. Results of Kruskal-Wallis H test for statistically significant differences of polar lipid classes between the groups. Abbreviations: monogalactosyl diacylglycerol–MGDG, digalactosyl diacylglycerol–DGDG, sulfoquinovosyl diacylglycerol–SQDG, phosphatidylcholine–PC, lyso phosphatidylcholine–LPC, phosphatidylethanolamine–PE, phosphatidylglycerol–PG, phosphatidylinositol–PI, diacylglyceryl-O-4′-(N,N,N-trimethyl) homoserine–DGTS, Table S4. Results of post hoc Dunn’s multiple comparison test. Statistically significant lipid differences between the groups are highlighted in bold. Abbreviations: monogalactosyl diacylglycerol–MGDG, digalactosyl diacylglycerol–DGDG, sulfoquinovosyl diacylglycerol–SQDG, phosphatidylcholine–PC, lyso phosphatidylcholine–LPC, phosphatidylethanolamine–PE, phosphatidylglycerol–PG, phosphatidylinositol–PI, diacylglyceryl-O-4′-(N,N,N-trimethyl) homoserine–DGTS, Table S5. Main polar lipid species contributors for the PCA first dimension. Abbreviations: monogalactosyl diacylglycerol–MGDG, monogalactosyl monoacylglycerol–MGMG, sulfoquinovosyl diacylglycerol–SQDG, sulfoquinovosyl monoacylglycerol–SQMG, phosphatidylglycerol–PG, phosphatidylinositol–PI, monoacylglyceryl-O-4′-(N,N,N-trimethyl) homoserine–MGTS, Table S6. Results of Kruskal-Wallis H test for statistically significant differences of polar lipid species between the groups. Abbreviations: monogalactosyl diacylglycerol–MGDG, monogalactosyl monoacylglycerol–MGMG, digalactosyl diacylglycerol–DGDG, digalactosyl monoacylglycerol–DGMG, sulfoquinovosyl diacylglycerol–SQDG, sulfoquinovosyl monoacylglycerol–SQMG, phosphatidylcholine–PC, lyso phosphatidylcholine–LPC, phosphatidylethanolamine–PE, phosphatidylglycerol–PG, phosphatidylinositol–PI, phosphoinositol ceramide–PI-Cer, diacylglyceryl-O-4′-(N,N,N-trimethyl) homoserine–DGTS, monoacylglyceryl-O-4′-(N,N,N-trimethyl) homoserine–MGTS.

## References

[1] Ma X.-N., Chen T.-P., Yang B., Liu J., Chen F. (2016). Lipid Production from Nannochloropsis. Mar. Drugs.

[2] Mitra M., Patidar S.K., Mishra S. (2015). Integrated process of two stage cultivation of Nannochloropsis sp. for nutraceutically valuable eicosapentaenoic acid along with biodiesel. Bioresour. Technol..

[3] Miles E., Childs C., Calder P. (2021). Long-Chain Polyunsaturated Fatty Acids (LCPUFAs) and the Developing Immune System: A Narrative Review. Nutrients.

[4] Meng Y., Cao X., Yao C., Xue S., Yang Q. (2017). Identification of the role of polar glycerolipids in lipid metabolism and their acyl attribution for TAG accumulation in Nannochloropsis oceanica. Algal Res..

[5] Wang X., Fosse H.K., Li K., Chauton M.S., Vadstein O., Reitan K.I. (2019). Influence of Nitrogen Limitation on Lipid Accumulation and EPA and DHA Content in Four Marine Microalgae for Possible Use in Aquafeed. Front. Mar. Sci..

[6] Martin G.J.O., Hill D.R.A., Olmstead I.L.D., Bergamin A., Shears M., Dias D.A., Kentish S.E., Scales P.J., Botté C.Y., Callahan D.L. (2014). Lipid Profile Remodeling in Response to Nitrogen Deprivation in the Microalgae Chlorella sp. (Trebouxiophyceae) and Nannochloropsis sp. (Eustigmatophyceae). PLoS ONE.

[7] Ryckebosch E., Bruneel C., Termote-Verhalle R., Goiris K., Muylaert K., Foubert I. (2014). Nutritional evaluation of microalgae oils rich in omega-3 long chain polyunsaturated fatty acids as an alternative for fish oil. Food Chem..

[8] Lordan R., Tsoupras A., Zabetakis I. (2017). Phospholipids of Animal and Marine Origin: Structure, Function, and Anti-Inflammatory Properties. Molecules.

[9] Andrianasolo E.H., Haramaty L., Vardi A., White E., Lutz R., Falkowski P. (2008). Apoptosis-Inducing Galactolipids from a Cultured Marine Diatom, Phaeodactylum tricornutum. J. Nat. Prod..

[10] Morimoto T., Nagatsu A., Murakami N., Sakakibara J., Tokuda H., Nishino H., Iwashima A. (1995). Anti-tumour-promoting glyceroglycolipids from the green alga, Chlorella vulgaris. Phytochemistry.

[11] Chirasuwan N., Chaiklahan R., Kittakoop P., Chanasattru W., Ruengjitchatchawalya M., Tanticharoen M., Bunnag B. (2009). Anti HSV-1 activity of sulphoquinovosyl diacylglycerol isolated from Spirulina platensis. Sci. Asia.

[12] Reshef V., Mizrachi E., Maretzki T., Silberstein C., Loya S., Hizi A., Carmeli S. (1997). New Acylated Sulfoglycolipids and Digalactolipids and Related Known Glycolipids from Cyanobacteria with a Potential To Inhibit the Reverse Transcriptase of HIV-1. J. Nat. Prod..

[13] Ahamed A.A.P., Rasheed M.U., Noorani P.M., Reehana N., Santhoshkumar S., Imran Y.M.M., Alharbi N.S., Arunachalam C., Alharbi S.A., Akbarsha M.A. (2017). In vitro antibacterial activity of MGDG-palmitoyl from Oscillatoria acuminata NTAPC05 against extended-spectrum β-lactamase producers. J. Antibiot..

[14] Banskota A.H., Stefanova R., Gallant P., McGinn P.J. (2013). Mono- and digalactosyldiacylglycerols: Potent nitric oxide inhibitors from the marine microalga Nannochloropsis granulata. Environ. Boil. Fishes.

[15] Banskota A.H., Gallant P., Stefanova R., Melanson R., O’Leary S.J.B. (2013). Monogalactosyldiacylglycerols, potent nitric oxide inhibitors from the marine microalgaTetraselmis chui. Nat. Prod. Res..

[16] Bruno A., Rossi C., Marcolongo G., Di Lena A., Venzo A., Berrie C.P., Corda D. (2005). Selective in vivo anti-inflammatory action of the galactolipid monogalactosyldiacylglycerol. Eur. J. Pharmacol..

[17] Banskota A.H., Stefanova R., Sperker S., Melanson R., Osborne J.A., O’Leary S.J.B. (2013). Five new galactolipids from the freshwater microalga Porphyridium aerugineum and their nitric oxide inhibitory activity. Environ. Boil. Fishes.

[18] Sun N., Chen J., Wang D., Lin S. (2018). Advance in food-derived phospholipids: Sources, molecular species and structure as well as their biological activities. Trends Food Sci. Technol..

[19] Traversier M., Gaslondes T., Milesi S., Michel S., Delannay E. (2018). Polar lipids in cosmetics: Recent trends in extraction, separation, analysis and main applications. Phytochem. Rev..

[20] Zanella L., Vianello F. (2020). Microalgae of the genus Nannochloropsis: Chemical composition and functional implications for human nutrition. J. Funct. Foods.

[21] Callejón M.J.J., Medina A.R., Moreno P.A.G., Cerdán L.E., Guillén S.O., Grima E.M. (2020). Simultaneous extraction and fractionation of lipids from the microalga Nannochloropsis sp. for the production of EPA-rich polar lipid concentrates. Environ. Boil. Fishes.

[22] Couto D., Melo T., Conde T.A., Costa M., Silva J., Domingues M.R.M., Domingues P. (2021). Chemoplasticity of the polar lipid profile of the microalgae Chlorella vulgaris grown under heterotrophic and autotrophic conditions. Algal Res..

[23] Conde T., Neves B., Couto D., Melo T., Neves B., Costa M., Silva J., Domingues P., Domingues M. (2021). Microalgae as Sustainable Bio-Factories of Healthy Lipids: Evaluating Fatty Acid Content and Antioxidant Activity. Mar. Drugs.

[24] Conde T.A., Couto D., Melo T., Costa M., Silva J., Domingues M.R., Domingues P. (2021). Polar lipidomic profile shows Chlorococcum amblystomatis as a promising source of value-added lipids. Sci. Rep..

[25] Munteanu I.G., Apetrei C. (2021). Analytical Methods Used in Determining Antioxidant Activity: A Review. Int. J. Mol. Sci..

[26] Koyande A.K., Chew K.W., Rambabu K., Tao Y., Chu D.-T., Show P.-L. (2019). Microalgae: A potential alternative to health supplementation for humans. Food Sci. Hum. Wellness.

[27] Mercer P., Armenta R.E. (2011). Developments in oil extraction from microalgae. Eur. J. Lipid Sci. Technol..

[28] Folch J., Lees M., Sloane Stanley G.H. (1957). A Simple Method for the Isolation and Purification of Total Lipides from Animal Tissues. J. Biol. Chem..

[29] European Commission (2010). Directive 2009/32/EC of the European Parliament and of the Council of 23 April 2009 on the Approximation of the Laws of the Member States on Extraction Solvents Used in the Production of Foodstuffs and Food Ingredients.

[30] Michalak I., Chojnacka K. (2014). Algal extracts: Technology and advances. Eng. Life Sci..

[31] Chemat F., Rombaut N., Sicaire A.-G., Meullemiestre A., Fabiano-Tixier A.-S., Abert-Vian M. (2017). Ultrasound assisted extraction of food and natural products. Mechanisms, techniques, combinations, protocols and applications. A review. Ultrason. Sonochem..

[32] Figueiredo A., da Costa E., Silva J., Domingues M.R., Domingues P. (2019). The effects of different extraction methods of lipids from Nannochloropsis oceanica on the contents of omega-3 fatty acids. Algal Res..

[33] Mitra M., Mishra S. (2019). A comparative analysis of different extraction solvent systems on the extractability of eicosapentaenoic acid from the marine eustigmatophyte Nannochloropsis oceanica. Algal Res..

[34] Ryckebosch E., Bruneel C., Termote-Verhalle R., Muylaert K., Foubert I. (2014). Influence of extraction solvent system on extractability of lipid components from different microalgae species. Algal Res..

[35] Maciel E., Leal M.C., Lillebø A.I., Domingues P., Domingues M.R., Calado R. (2016). Bioprospecting of Marine Macrophytes Using MS-Based Lipidomics as a New Approach. Mar. Drugs.

[36] Han D., Jia J., Li J., Sommerfeld M., Xu J., Hu Q. (2017). Metabolic Remodeling of Membrane Glycerolipids in the Microalga Nannochloropsis oceanica under Nitrogen Deprivation. Front. Mar. Sci..

[37] Willette S., Gill S.S., Dungan B., Schaub T.M., Jarvis J.M., Hilaire R.S., Holguin F.O. (2018). Alterations in lipidome and metabolome profiles of Nannochloropsis salina in response to reduced culture temperature during sinusoidal temperature and light. Algal Res..

[38] He H., Rodgers R.P., Marshall A.G., Hsu C.S. (2011). Algae Polar Lipids Characterized by Online Liquid Chromatography Coupled with Hybrid Linear Quadrupole Ion Trap/Fourier Transform Ion Cyclotron Resonance Mass Spectrometry. Energy Fuels.

[39] Cutignano A., Luongo E., Nuzzo G., Pagano D., Manzo E., Sardo A., Fontana A. (2016). Profiling of complex lipids in marine microalgae by UHPLC/tandem mass spectrometry. Algal Res..

[40] Li S., Xu J., Jiang Y., Zhou C., Yu X., Zhong Y., Chen J., Yan X. (2015). Lipidomic analysis can distinguish between two morphologically similar strains of Nannochloropsis oceanica. J. Phycol..

[41] Gorgich M., Mata T., Martins A., Vieira M.B., Caetano N. (2020). Comparison of different lipid extraction procedures applied to three microalgal species. Energy Rep..

[42] Breil C., Vian M.A., Zemb T., Kunz W., Chemat F. (2017). “Bligh and Dyer” and Folch Methods for Solid–Liquid–Liquid Extraction of Lipids from Microorganisms. Comprehension of Solvatation Mechanisms and towards Substitution with Alternative Solvents. Int. J. Mol. Sci..

[43] Cequier-Sánchez E., Rodríguez C., Ravelo Á.G., Zárate R. (2008). Dichloromethane as a Solvent for Lipid Extraction and Assessment of Lipid Classes and Fatty Acids from Samples of Different Natures. J. Agric. Food Chem..

[44] Li Y., Naghdi F.G., Garg S., Adarme-Vega T.C., Thurecht K.J., Ghafor W.A., Tannock S., Schenk P.M. (2014). A comparative study: The impact of different lipid extraction methods on current microalgal lipid research. Microb. Cell Factories.

[45] Stranska-Zachariasova M., Kastanek P., Dzuman Z., Rubert J., Godula M., Hajslova J. (2016). Bioprospecting of microalgae: Proper extraction followed by high performance liquid chromatographic–high resolution mass spectrometric fingerprinting as key tools for successful metabolom characterization. J. Chromatogr. B.

[46] Ryckebosch E., Bermúdez S.P.C., Termote-Verhalle R., Bruneel C., Muylaert K., Parra-Saldivar R., Foubert I. (2014). Influence of extraction solvent system on the extractability of lipid components from the biomass of Nannochloropsis gaditana. Environ. Boil. Fishes.

[47] Simopoulos A.P. (2008). The Importance of the Omega-6/Omega-3 Fatty Acid Ratio in Cardiovascular Disease and Other Chronic Diseases. Exp. Biol. Med..

[48] Burri L., Hoem N., Banni S., Berge K. (2012). Marine Omega-3 Phospholipids: Metabolism and Biological Activities. Int. J. Mol. Sci..

[49] Banskota A.H., Stefanova R., Sperker S., Lall S.P., Craigie J.S., Hafting J.T., Critchley A. (2014). Polar lipids from the marine macroalga Palmaria palmata inhibit lipopolysaccharide-induced nitric oxide production in RAW264.7 macrophage cells. Phytochemistry.

[50] van Hoogevest B.P., Prusseit R.W. (2013). Phospholipids: Natural Functional Ingredients and Actives for Cosmetic Products. SOFW-J..

[51] Li J., Wang X., Zhang T., Wang C., Huang Z., Luo X., Deng Y. (2015). A review on phospholipids and their main applications in drug delivery systems. Asian J. Pharm. Sci..

[52] da Costa E., Silva J., Mendonça S.H., Abreu M.H., Domingues M.R. (2016). Lipidomic Approaches towards Deciphering Glycolipids from Microalgae as a Reservoir of Bioactive Lipids. Mar. Drugs.

[53] Banskota A.H., Stefanova R., Sperker S., McGinn P.J. (2013). New diacylglyceryltrimethylhomoserines from the marine microalga Nannochloropsis granulata and their nitric oxide inhibitory activity. Environ. Boil. Fishes.

[54] Khatib S., Artoul F., Paluy I., Boluchevsky L., Kvitnitsky E., Vaya J. (2017). Nannochloropsis sp. ethanol extract prevents macrophage and LDL oxidation and enhances PON1 activity through the principal active compound lyso-diacylglyceryltrimethylhomoserine (lyso-DGTS). Environ. Boil. Fishes.

[55] Chemat F., Khan M.K. (2011). Applications of ultrasound in food technology: Processing, preservation and extraction. Ultrason. Sonochem..

[56] Cena H., Calder P.C. (2020). Defining a Healthy Diet: Evidence for the Role of Contemporary Dietary Patterns in Health and Disease. Nutrients.

[57] Guerra I., Pereira H., Costa M., Silva J., Santos T., Varela J., Mateus M., Silva J. (2021). Operation Regimes: A Comparison Based on *Nannochloropsis oceanica* Biomass and Lipid Productivity. Energies.

[58] Colombo S., Melo T., Martínez-López M., Carrasco M.J., Domingues M.R., Pérez-Sala D., Domingues P. (2018). Phospholipidome of endothelial cells shows a different adaptation response upon oxidative, glycative and lipoxidative stress. Sci. Rep..

[59] Anjos S., Feiteira E., Cerveira F., Melo T., Reboredo A., Colombo S., Dantas R., Costa E., Moreira A., Santos S. (2019). Lipidomics Reveals Similar Changes in Serum Phospholipid Signatures of Overweight and Obese Pediatric Subjects. J. Proteome Res..

[60] Pluskal T., Castillo S., Villar-Briones A., Orešič M. (2010). MZmine 2: Modular framework for processing, visualizing, and analyzing mass spectrometry-based molecular profile data. BMC Bioinform..

[61] The LIPID MAPS Lipidomics Gateway. http://www.lipidmaps.org/.

[62] Da Costa E., Amaro H.M., Melo T., Guedes A.C., Domingues M.R. (2020). Screening for polar lipids, antioxidant, and anti-inflammatory activities of Gloeothece sp. lipid extracts pursuing new phytochemicals from cyanobacteria. Environ. Boil. Fishes.

[63] Da Costa E., Melo T., Moreira A.S.P., Bernardo C., Helguero L., Ferreira I., Cruz M.T., Rego A.M., Domingues P., Calado R. (2017). Valorization of Lipids from Gracilaria sp. through Lipidomics and Decoding of Antiproliferative and Anti-Inflammatory Activity. Mar. Drugs.

[64] Liebisch G., Fahy E., Aoki J., Dennis E.A., Durand T., Ejsing C.S., Fedorova M., Feussner I., Griffiths W.J., Köfeler H. (2020). Update on LIPID MAPS classification, nomenclature, and shorthand notation for MS-derived lipid structures. J. Lipid Res..

[65] Melo T., Marques S., Ferreira I., Cruz M.T., Domingues P., Segundo M., Domingues M.R.M. (2018). New Insights into the Anti-Inflammatory and Antioxidant Properties of Nitrated Phospholipids. Lipids.

[66] R Core Team (2018). R: A Language and Environment for Statistical Computing.

[67] Rstudio Team (2016). Rstudio: Integrated Development Environment for R. RStudio, PBC.

[68] Karpievitch Y.V., Nikolic S.B., Wilson R., Sharman J., Edwards L.M. (2014). Metabolomics Data Normalization with EigenMS. PLoS ONE.

[69] Xia J., Wishart D.S. (2016). Using MetaboAnalyst 3.0 for Comprehensive Metabolomics Data Analysis. Curr. Protoc. Bioinform..

[70] Stacklies W., Redestig H., Scholz M., Walther D., Selbig J. (2007). pcaMethods a bioconductor package providing PCA methods for incomplete data. Bioinformatics.

[71] Benjamini Y., Hochberg Y. (1995). Controlling the False Discovery Rate: A Practical and Powerful Approach to Multiple Testing. J. R. Stat. Soc. Ser. B.

[72] Wickham H. (2016). Ggplot2—Elegant Graphics for Data Analysis.

