# Supplementary material for: Ethanol Extraction of Polar Lipids from Nannochloropsis oceanica for Food, Feed, and Biotechnology Applications Evaluated Using Lipidomic Approaches
Source: Mar Drugs. 2021 Oct 21;19(11):593. doi: 10.3390/md19110593 (PMC8624173; doi:10.3390/md19110593)
Supplement: Supplementary file 1 [file marinedrugs-19-00593-s001.zip › marinedrugs-1404782-supplementary.pdf]

Ethanol Extraction of Polar Lipids from *Nannochloropsis oceanica* for Food, Feed, and Biotechnology Applications Evaluated Using Lipidomic Approaches

Tânia Melo [1,2,*,†], Ana R. P. Figueiredo [1,†], Elisabete da Costa [1,2], Daniela Couto [1,2], Joana Silva [3], M. Rosário Domingues [1,2] and Pedro Domingues [1,*]

[1] Mass Spectrometry Center, LAQV-REQUIMTE, Department of Chemistry, University of Aveiro,
Santiago University Campus, 3810-193 Aveiro, Portugal; ana90@ua.pt (A.R.P.F.); elisabetecosta@ua.pt (E.d.C.); danielacouto@ua.pt (D.C.); mrd@ua.pt (M.R.D.)

[2] CESAM-Centre for Environmental and Marine Studies, Department of Chemistry, University of Aveiro, Santiago University Campus, 3810-193 Aveiro, Portugal

[3] Allmicroalgae—Natural Products S.A., Avenida das Forças Armadas, 125, 7º piso, 1600-079 Lisboa, Portugal; joana.g.silva@allmicroalgae.com

* Correspondence: taniamelo@ua.pt (T.M.); p.domingues@ua.pt (P.D.)

† Both contributed equally to the manuscript.

Supplementary Table S1. Polar lipid species identified in the lipidome of *Nannochloropsis oceanica* by HILIC–MS and MS/MS, with information on the type of ion, lipid identity, calculated mass, observed mass, mass error (Δ ppm), fatty acyl composition, and molecular formula. The glycolipid species belonging to MGDG, MGMG, DGDG, and DGMG classes were identified as $[M + NH_4]^+$ ions; SQDG and SQMG were identified as $[M – H]^−$ ions. Phospholipid species of PC, LPC, and PE as well as betaine lipids from DGTS and MGTS classes were identified as $[M + H]^+$ ions while the lipid species from phospholipid classes PG and PI and sphingolipids PI-Cer were identified as $[M – H]^−$ ions. In the fatty acyl chains, C represents the total number of carbon atoms and N represents the total number of double bonds. The C:N/C:N assignment indicates the attribution of fatty acyl chains to the position *sn*-1/*sn*-2, while in C:N_C:N assignment the attribution of the *sn*-1 and *sn*-2 position of fatty acyl chains is not known. * Assignments by mass accuracy, unequivocal molecular formula, and typical retention time of lipid class, but without MS/MS.

**Lipid species (C:N)**	**Calculated *m/z***	**Observed *m/z***	**Error (ppm)**	**Fatty acyl chains (C:N)**	**Formula**
**Glycolipid species**					
**MGDG identified as $[M + NH_4]^+$**					
MGDG(30:1)	718.5464	718.5470	0.8350	16:1_14:0	C39H76NO10
MGDG(32:1)	746.5777	746.5788	1.4734	16:1_16:0	C41H80NO10
MGDG(32:2)	744.5626	744.5620	-0.8058	16:1/16:1	C41H78NO10
MGDG(32:3)	742.5464	742.5469	0.6734	16:2_16:1	C41H76NO10
MGDG(32:5)	738.5156	738.5157	0.1029	20:5_12:0	C41H72NO10
MGDG(34:2)	772.5933	772.5936	0.3883	16:1_18:1	C43H82NO10
MGDG(34:5)	766.5469	766.5459	-1.3359	20:5_14:0	C43H76NO10
MGDG(36:5)	794.5782	794.5781	-0.1259	20:5_16:0	C45H80NO10
MGDG(36:6)	792.5625	792.5626	0.1262	20:5_16:1	C45H78NO10
MGDG(38:5)	822.6095	822.6096	0.0973	*	C47H84NO10
MGDG(38:6)	820.5939	820.5934	-0.6093	*	C47H82NO10
MGDG(38:7)	818.5782	818.5792	1.1923	*	C47H80NO10
MGDG(38:8)	816.5626	816.5613	-1.5553	20:5_18:3	C47H78NO10
MGDG(40:8)	844.5939	844.5936	-0.3697	20:4/20:4	C49H82NO10
MGDG(40:10)	840.5626	840.5631	0.6840	20:5/20:5	C49H78NO10
**MGMG identified as $[M + NH_4]^+$**					
MGMG(14:0)	482.3329	482.3325	-0.8480	*	C23H48NO9

MGMG(16:0)	510.3642	510.3638	-0.8014	*	C25H52NO9
MGMG(16:1)	508.3486	508.3486	0.0807	*	C25H50NO9
MGMG(20:4)	558.3642	558.3622	-3.5980	*	C29H52NO9
MGMG(20:5)	556.3486	556.3488	0.4332	20:5	C29H50NO9
**DGDG identified as [M + NH_4]$^+$**					
DGDG(30:1)	880.5997	880.6003	0.6351	16:1_14:0	C45H86O15N
DGDG(32:1)	908.6310	908.6309	-0.1640	16:1_16:0	C47H90O15N
DGDG(32:2)	906.6154	906.6160	0.6629	16:1/16:1	C47H88O15N
DGDG(32:5)	900.5684	900.5698	1.5002	*	C47H82O15N
DGDG(34:1)	936.6623	936.6590	-3.5755	18:1_16:0	C49H94O15N
DGDG(34:2)	934.6467	934.6466	-0.1059	18:2_16:0; 18:1_16:1	C49H92O15N
DGDG(34:3)	932.6310	932.6320	1.0197	18:2_16:1	C49H90O15N
DGDG(34:4)	930.6154	930.6121	-3.5450	*	C49H88O15N
DGDG(34:5)	928.5997	928.6011	1.4549	20:5_14:0	C49H86O15N
DGDG(36:5)	956.6310	956.6306	-0.4181	20:5_16:0	C51H90O15N
DGDG(36:6)	954.6154	954.6161	0.7343	20:5_16:1	C51H88O15N
DGDG(36:7)	952.5997	952.5999	0.1585	20:5_16:2	C51H86O15N
DGDG(38:7)	980.6310	980.6308	-0.2539	*	C53H90O15N
DGDG(40:10)	1002.6154	1002.6119	-3.4899	20:5_20:5	C55H88O15N
**DGMG identified as [M + NH_4]$^+$**					
DGMG(16:1)	670.4014	670.4018	0.6099	16:1	C31H60NO14
DGMG(16:0)	672.4170	672.4171	0.1148	16:0	C31H62NO14
**SQDG identified as [M − H]$^-$**					
SQDG(28:1)	735.4353	735.4358	0.6432	*	C37H67O12S
SQDG(30:1)	763.4666	763.4669	0.3664	16:1_14:0	C39H71O12S
SQDG(30:2)	761.4510	761.4514	0.5555	*	C39H69O12S
SQDG(31:1)	777.4823	777.4827	0.5441	15:0_16:1	C40H73O12S
SQDG(32:1)	791.4979	791.4990	1.3557	16:1_16:0	C41H75O12S
SQDG(32:2)	789.4823	789.4830	0.9158	16:1/16:1	C41H73O12S
SQDG(32:3)	787.4666	787.4679	1.6166	*	C41H71O12S
SQDG(34:1)	819.5292	819.5307	1.7974	18:1_16:0	C43H79O12S
SQDG(34:2)	817.5136	817.5156	2.4758	18:2_16:0	C43H77O12S
SQDG(36:5)	839.4979	839.4969	-1.2234	*	C45H75O12S
**SQMG identified as [M − H]$^-$**					
SQMG(16:0)	555.2839	555.2854	2.6797	*	C25H47O11S
**Phospholipid species**					
**PC identified as [M + H]$^+$**					
PC(30:0)	706.5387	706.5354	-4.6452	14:0_16:0	C38H77NO8P
PC(30:1)	704.5230	704.5236	0.8062	14:0_16:1	C38H75NO8P
PC(32:2)	730.5387	730.5387	-0.0264	16:1/16:1	C40H77NO8P
PC(32:3)	728.5230	728.5231	0.0933	16:1_16:2	C40H75NO8P
PC(34:2)	758.5700	758.5694	-0.7672	16:1_18:1	C42H81NO8P
PC(34:3)	756.5543	756.5545	0.2234	16:1_18:2	C42H79NO8P
PC(34:4)	754.5387	754.5377	-1.3015	16:1_18:3; 16:2_18:2	C42H77NO8P
PC(36:2)	786.6013	786.6000	-1.6298	18:1/18:1	C44H85NO8P
PC(36:3)	784.5856	784.5849	-0.9330	18:1_18:2	C44H83NO8P
PC(36:4)	782.5700	782.5686	-1.7660	18:2/18:2; 16:0_20:4	C44H81NO8P

PC(36:5)	780.5543	780.5530	-1.7052	16:1_20:4	C44H79NO8P
PC(36:6)	778.5387	778.5378	-1.1329	16:1_20:5	C44H77NO8P
PC(36:7)	776.5230	776.5211	-2.4880	16:2_20:5	C44H75NO8P
PC(38:5)	808.5856	808.5851	-0.6579	*	C46H83NO8P
PC(38:6)	806.5700	806.5694	-0.7216	18:1_20:5; 18:2_20:4	C46H81NO8P
PC(38:7)	804.5543	804.5528	-1.9029	18:2_20:5; 18:3_20:4	C46H79NO8P
PC(38:8)	802.5387	802.5360	-3.3513	18:3_20:5	C46H77NO8P
PC(38:9)	800.5230	800.5207	-2.9131	*	C46H75NO8P
PC(40:9)	828.5543	828.5519	-2.9340	20:4_20:5	C48H79NO8P
PC(40:10)	826.5387	826.5387	0.0218	20:5/20:5	C48H77NO8P
**LPC identified as $[M + H]^+$**					
LPC(14:0)	468.3090	468.3072	-3.8799	14:0	C22H47NO7P
LPC(16:0)	496.3403	496.3406	0.5722	16:0	C24H51NO7P
LPC(16:1)	494.3247	494.3245	-0.3378	16:1	C24H49NO7P
LPC(16:2)	492.3090	492.3088	-0.4408	16:2	C24H47NO7P
LPC(18:1)	522.3560	522.3564	0.8289	18:1	C26H53NO7P
LPC(18:2)	520.3403	520.3405	0.3536	18:2	C26H51NO7P
LPC(18:3)	518.3247	518.3232	-2.8303	18:3	C26H49NO7P
LPC(20:4)	544.3403	544.3391	-2.2339	20:4	C28H51NO7P
LPC(20:5)	542.3247	542.3237	-1.7831	20:5	C28H49NO7P
**PE identified as $[M + H]^+$**					
PE(30:1)	662.4761	662.4763	0.3291	*	C35H69NO8P
PE(32:1)	690.5074	690.5091	2.3432	*	C37H73NO8P
PE(32:2)	688.4917	688.4919	0.2440	16:1_16:1	C37H71O8NP
PE(34:2)	716.5230	716.5221	-1.2800	*	C39H77NO8P
PE(36:5)	738.5074	738.5105	4.2220	16:1_20:4	C41H73O8NP
PE(36:6)	736.4917	736.4920	0.3639	16:1_20:5	C41H71NO8P
PE(38:6)	764.5230	764.5219	-1.4807	18:2/20:4	C43H75NO8P
PE(40:8)	788.5230	788.5243	1.6080	*	C45H75O8NP
PE(40:9)	786.5074	786.5059	-1.8840	20:4_20:5	C45H73O8NP
PE(40:10)	784.4917	784.4904	-1.6980	*	C45H71NO8P
**PG identified as $[M - H]^-$**					
PG(30:0)	693.4707	693.4710	0.4326	*	C36H70O10P
PG(30:1)	691.4550	691.4555	0.7231	14:0_16:1	C36H68O10P
PG(32:1)	719.4863	719.4871	1.1119	16:0_16:1	C38H72O10P
PG(32:2)	717.4707	717.4712	0.6969	16:1/16:1	C38H70O10P
PG(34:1)	747.5176	747.5182	0.8027	16:0_18:1	C40H76O10P
PG(34:2)	745.5020	745.5029	1.2072	16:0_18:2<br>16:1_18:1	C40H74O10P
PG(34:3)	743.4863	743.4867	0.5380	*	C40H72O10P
PG(34:5)	739.4550	739.4550	0.0000	14:0_20:5	C40H68O10P
PG(36:2)	773.5333	773.5342	1.1635	*	C42H78O10P
PG(36:5)	767.4863	767.4871	1.0254	16:0_20:5	C42H72O10P
PG(36:6)	765.4707	765.4728	2.7917	16:1_20:5	C42H70O10P
**PI identified as $[M - H]^-$**					
PI(30:1)	779.4711	779.4700	-1.4112	14:0_16:1	C39H72O13P
PI(32:1)	807.5024	807.5031	0.8669	16:0_16:1	C41H76O13P
PI(32:2)	805.4867	805.4843	-2.9796	16:1/16:1	C41H74O13P

PI(34:2)	833.5180	833.5180	0.0000	16:1_18:1; 16:0_18:2	C43H78O13P
PI(34:3)	831.5024	831.5023	-0.1203	16:0_18:3<br>16:1_18:2	C43H76O13P
PI(36:5)	855.5024	855.4983	-4.7925	*	C45H76O13P
**Sphingolipid species**					
**PI-Cer identified as [M − H]$^-$**					
PI-Cer (d32:1)	750.4921	750.4928	0.8967	d18:1/14:0	C38H73NO11P
PI-Cer (d32:2)	748.4765	748.4772	0.9673	d18:1/14:1	C38H71NO11P
**Betaine Lipid species**					
**DGTS identified as [M + H]$^+$**					
DGTS(30:1)	682.5622	682.5621	-0.1157	14:0_16:1	C40H76O7N
DGTS(32:1)	710.5935	710.5929	-0.8148	*	C42H80O7N
DGTS(32:2)	708.5778	708.5777	-0.1821	16:1/16:1	C42H78O7N
DGTS(32:3)	706.5622	706.5617	-0.6779	16:1_16:2	C42H76O7N
DGTS(34:1)	738.6248	738.6236	-1.5962	16:0/18:1	C44H84O7N
DGTS(34:2)	736.6091	736.6091	-0.0380	16:1_18:1	C44H82O7N
DGTS(34:3)	734.5935	734.5930	-0.6521	16:1_18:2	C44H80O7N
DGTS(34:4)	732.5778	732.5755	-3.1792	*	C44H78O7N
DGTS(34:5)	730.5622	730.5613	-1.2032	14:0_20:5	C44H76O7N
DGTS(36:4)	760.6091	760.6072	-2.5348	*	C46H82O7N
DGTS(36:5)	758.5935	758.5922	-1.6860	*	C46H80O7N
DGTS(36:6)	756.5778	756.5778	-0.0383	16:1_20:5	C46H78O7N
DGTS(36:7)	754.5622	754.5619	-0.3698	16:2_20:5	C46H76O7N
DGTS(38:5)	786.6248	786.6218	-3.7871	*	C48H84O7N
DGTS(38:6)	784.6091	784.6082	-1.1828	*	C48H82O7N
DGTS(38:7)	782.5935	782.5930	-0.6121	18:2_20:5	C48H80O7N
DGTS(38:8)	780.5778	780.5768	-1.3183	18:3_20:5	C48H78O7N
DGTS(40:9)	806.5935	806.5906	-3.5693	*	C50H80O7N
DGTS(40:10)	804.5778	804.5781	0.3368	20:5/20:5	C50H78O7N
**MGTS identified as [M + H]$^+$**					
MGTS(16:0)	474.3795	474.3795	0.0759	16:0	C26H52O6N
MGTS(16:1)	472.3638	472.3631	-1.5115	16:1	C26H50O6N
MGTS(20:4)	522.3795	522.3797	0.4518	*	C30H52O6N
MGTS(20:5)	520.3638	520.3635	-0.6034	20:5	C30H50O6N

Abbreviations: monogalactosyl diacylglycerol – MGDG, monogalactosyl monoacylglycerol – MGMG, digalactosyl diacylglycerol – DGDG, digalactosyl monoacylglycerol – DGMG, sulfoquinovosyl diacylglycerol – SQDG, sulfoquinovosyl monoacylglycerol – SQMG, phosphatidylcholine – PC, lyso phosphatidylcholine – LPC, phosphatidylethanolamine – PE, phosphatidylglycerol – PG, phosphatidylinositol – PI, phosphoinositol ceramide – PI-Cer, diacylglyceryl-O-4′-(N,N,N-trimethyl) homoserine – DGTS, monoacylglyceryl-O-4′-(N,N,N-trimethyl) homoserine – MGTS.

Supplementary Table S2. Main contributors for the PCA first dimension allowing discrimination between the groups based on polar lipid classes. Abbreviations: monogalactosyl diacylglycerol – MGDG, digalactosyl diacylglycerol – DGDG, sulfoquinovosyl diacylglycerol – SQDG, phosphatidylcholine – PC, lyso phosphatidylcholine – LPC, phosphatidylethanolamine – PE, phosphatidylglycerol – PG, phosphatidylinositol – PI, diacylglyceryl-O-4′-(N,N,N-trimethyl) homoserine – DGTS.

Label	Dim. 1(%)
**SGDG**	15.2305
**PC**	14.9109
**PG**	14.7652
**DGDG**	14.2460
**PE**	13.3451
**LPC**	11.7548
**PI**	11.5757
**DGTS**	2.6550
**MGDG**	1.5169

Supplementary Table S3. Results of Kruskal-Wallis H test for statistically significant differences of polar lipid classes between the groups. Abbreviations: monogalactosyl diacylglycerol – MGDG, digalactosyl diacylglycerol – DGDG, sulfoquinovosyl diacylglycerol – SQDG, phosphatidylcholine – PC, lyso phosphatidylcholine – LPC, phosphatidylethanolamine – PE, phosphatidylglycerol – PG, phosphatidylinositol – PI, diacylglyceryl-O-4′-(N,N,N-trimethyl) homoserine – DGTS.

Label	K.W	Significance	FDR
**PI**	25.71	0.000102	0.000487
**PG**	25.57	0.000108	0.000487
**DGDG**	24.49	0.000174	0.000505
**SGDG**	23.92	0.000225	0.000505
**PC**	22.56	0.000410	0.000635
**PE**	22.49	0.000423	0.000635
**LPC**	21.36	0.000693	0.000891
**MGDG**	19.92	0.001292	0.001454
**DGTS**	17.13	0.004253	0.004253

Supplementary Table S4. Results of post hoc Dunn's multiple comparison test. Statistically significant lipid differences between the groups are highlighted in bold. Abbreviations: monogalactosyl diacylglycerol – MGDG, digalactosyl diacylglycerol – DGDG, sulfoquinovosyl diacylglycerol – SQDG, phosphatidylcholine – PC, lyso phosphatidylcholine – LPC, phosphatidylethanolamine – PE, phosphatidylglycerol – PG, phosphatidylinositol – PI, diacylglyceryl-O-4′-(N,N,N-trimethyl) homoserine – DGTS.

Label	P	P.adjusted	Comparisons
**LPC**	**0.00003**	**0.00048**	**Dichloromethane:ethanol - Ethanol+US Bath**
**PI**	**0.00006**	**0.00091**	**Dichloromethane:methanol - Ethanol**
**PG**	**0.00008**	**0.00114**	**Chloroform:methanol - Dichloromethane:ethanol**
**PG**	**0.00020**	**0.00153**	**Dichloromethane:ethanol - Dichloromethane:methanol**
**PI**	**0.00021**	**0.00160**	**Chloroform:methanol - Ethanol**
**PE**	**0.00013**	**0.00196**	**Dichloromethane:ethanol - Dichloromethane:methanol**
**SGDG**	**0.00014**	**0.00203**	**Chloroform:methanol - Dichloromethane:ethanol**
**PC**	**0.00018**	**0.00269**	**Chloroform:methanol - Dichloromethane:ethanol**
**DGDG**	**0.00043**	**0.00325**	**Chloroform:methanol - Ethanol**
**DGDG**	**0.00022**	**0.00331**	**Chloroform:methanol - Dichloromethane:ethanol**
**SGDG**	**0.00048**	**0.00361**	**Dichloromethane:ethanol - Dichloromethane:methanol**
**DGDG**	**0.00083**	**0.00415**	**Dichloromethane:ethanol - Dichloromethane:methanol**
**PE**	**0.00056**	**0.00422**	**Chloroform:methanol - Dichloromethane:ethanol**
**PI**	**0.00095**	**0.00474**	**Dichloromethane:ethanol - Dichloromethane:methanol**
**SGDG**	**0.00108**	**0.00541**	**Dichloromethane:ethanol - Ethanol+US Bath**
**DGTS**	**0.00036**	**0.00545**	**Dichloromethane:ethanol - Ethanol+US Bath**
**DGDG**	**0.00159**	**0.00598**	**Dichloromethane:methanol - Ethanol**
**PC**	**0.00088**	**0.00660**	**Chloroform:methanol - Ethanol**
**PG**	**0.00144**	**0.00720**	**Chloroform:methanol - Ethanol**
**PI**	**0.00239**	**0.00894**	**Chloroform:methanol - Dichloromethane:ethanol**
**MGDG**	**0.00181**	**0.00905**	**Dichloromethane:methanol - Ethanol**
**LPC**	**0.00140**	**0.01048**	**Chloroform:methanol - Dichloromethane:ethanol**
**MGDG**	**0.00073**	**0.01089**	**Dichloromethane:methanol - Ethanol+US Bath**
**PC**	**0.00232**	**0.01162**	**Dichloromethane:ethanol - Dichloromethane:methanol**
**MGDG**	**0.00181**	**0.01358**	**Dichloromethane:ethanol - Ethanol+US Bath**
**PG**	**0.00377**	**0.01413**	**Dichloromethane:methanol - Ethanol**
**PG**	**0.00476**	**0.01429**	**Dichloromethane:ethanol - Ethanol+US Bath**
**MGDG**	**0.00424**	**0.01590**	**Dichloromethane:ethanol - Ethanol**
**PE**	**0.00335**	**0.01673**	**Dichloromethane:methanol - Ethanol**
**SGDG**	**0.00509**	**0.01909**	**Chloroform:methanol - Ethanol**
**DGTS**	**0.00263**	**0.01971**	**Dichloromethane:methanol - Ethanol+US Bath**
**PC**	**0.00535**	**0.02005**	**Dichloromethane:ethanol - Ethanol+US Bath**
**LPC**	**0.00424**	**0.02120**	**Ethanol+US Bath - Ethanol+US probe**
**PE**	**0.00909**	**0.02727**	**Chloroform:methanol - Ethanol**
**PC**	**0.00933**	**0.02799**	**Dichloromethane:methanol - Ethanol**
**PE**	**0.00749**	**0.02811**	**Dichloromethane:ethanol - Ethanol+US Bath**
**PI**	**0.01283**	**0.03850**	**Ethanol - Ethanol+US Bath**
**SGDG**	**0.01578**	**0.04733**	**Dichloromethane:methanol - Ethanol**
**PC**	**0.01930**	**0.04824**	**Ethanol - Ethanol+US Bath**
**DGDG**	**0.01646**	**0.04938**	**Chloroform:methanol - Ethanol+US probe**
LPC	0.01746	0.05238	Dichloromethane:ethanol - Dichloromethane:methanol
DGDG	0.02130	0.05325	Dichloromethane:ethanol - Ethanol+US Bath
PG	0.02285	0.05712	Chloroform:methanol - Ethanol+US probe
SGDG	0.02285	0.05712	Chloroform:methanol - Ethanol+US probe
DGTS	0.01155	0.05777	Dichloromethane:ethanol - Ethanol
SGDG	0.02844	0.06094	Ethanol - Ethanol+US Bath
LPC	0.01746	0.06547	Dichloromethane:ethanol - Ethanol

DGDG	0.03427	0.07343	Ethanol - Ethanol+US Bath
DGTS	0.01989	0.07457	Chloroform:methanol - Ethanol+US Bath
PC	0.03565	0.07639	Chloroform:methanol - Ethanol+US probe
DGDG	0.04903	0.09192	Dichloromethane:methanol - Ethanol+US probe
PE	0.03755	0.09386	Dichloromethane:methanol - Ethanol+US probe
PI	0.03755	0.09386	Dichloromethane:methanol - Ethanol+US probe
PG	0.04491	0.09623	Ethanol - Ethanol+US Bath
PG	0.05345	0.10023	Dichloromethane:methanol - Ethanol+US probe
PI	0.05983	0.11218	Chloroform:methanol - Ethanol+US probe
PI	0.05345	0.11455	Ethanol - Ethanol+US probe
SGDG	0.06332	0.11872	Dichloromethane:methanol - Ethanol+US probe
LPC	0.04888	0.12221	Chloroform:methanol - Ethanol+US probe
DGTS	0.04903	0.12256	Dichloromethane:methanol - Ethanol
DGTS	0.04109	0.12326	Ethanol+US Bath - Ethanol+US probe
PG	0.07464	0.12440	Dichloromethane:ethanol - Ethanol+US probe
PI	0.07464	0.12440	Dichloromethane:ethanol - Ethanol+US Bath
PE	0.08089	0.13482	Dichloromethane:ethanol - Ethanol+US probe
PE	0.07464	0.13995	Ethanol - Ethanol+US Bath
LPC	0.07464	0.13995	Dichloromethane:methanol - Ethanol+US Bath
MGDG	0.05821	0.14553	Ethanol+US Bath - Ethanol+US probe
MGDG	0.04990	0.14969	Chloroform:methanol - Dichloromethane:methanol
PC	0.08089	0.15167	Dichloromethane:ethanol - Ethanol+US probe
PE	0.07135	0.15288	Chloroform:methanol - Ethanol+US probe
SGDG	0.10223	0.15335	Dichloromethane:ethanol - Ethanol+US probe
LPC	0.07464	0.15994	Ethanol - Ethanol+US Bath
SGDG	0.10223	0.17039	Ethanol+US Bath - Ethanol+US probe
MGDG	0.08621	0.18474	Chloroform:methanol - Dichloromethane:ethanol
MGDG	0.10223	0.19169	Ethanol - Ethanol+US probe
PI	0.12783	0.19175	Dichloromethane:methanol - Ethanol+US Bath
DGDG	0.12772	0.21287	Chloroform:methanol - Ethanol+US Bath
MGDG	0.13739	0.22899	Dichloromethane:methanol - Ethanol+US probe
PI	0.17483	0.23841	Chloroform:methanol - Ethanol+US Bath
DGDG	0.16940	0.25409	Dichloromethane:ethanol - Ethanol+US probe
DGTS	0.12783	0.27393	Dichloromethane:ethanol - Ethanol+US probe
PI	0.22035	0.27544	Dichloromethane:ethanol - Ethanol+US probe
PC	0.19365	0.29047	Dichloromethane:methanol - Ethanol+US probe
PG	0.19513	0.29269	Chloroform:methanol - Ethanol+US Bath
MGDG	0.22038	0.30051	Chloroform:methanol - Ethanol+US Bath
DGDG	0.23466	0.31999	Ethanol - Ethanol+US probe
PC	0.19365	0.32274	Ethanol - Ethanol+US probe
LPC	0.19365	0.32274	Dichloromethane:ethanol - Ethanol+US probe
MGDG	0.22035	0.33053	Dichloromethane:ethanol - Ethanol+US probe
DGTS	0.17763	0.33306	Chloroform:methanol - Ethanol
PC	0.26251	0.35797	Chloroform:methanol - Ethanol+US Bath
PC	0.29839	0.37299	Ethanol+US Bath - Ethanol+US probe
DGDG	0.29839	0.37299	Dichloromethane:methanol - Ethanol+US Bath
PE	0.24960	0.37441	Dichloromethane:methanol - Ethanol+US Bath
LPC	0.28147	0.38382	Dichloromethane:methanol - Ethanol+US probe

SGDG	0.28147	0.38382	Dichloromethane:ethanol - Ethanol
LPC	0.34000	0.39231	Chloroform:methanol - Dichloromethane:methanol
PG	0.29839	0.40689	Ethanol+US Bath - Ethanol+US probe
DGDG	0.35316	0.40749	Ethanol+US Bath - Ethanol+US probe
MGDG	0.32688	0.40860	Chloroform:methanol - Ethanol
PG	0.33424	0.41780	Ethanol - Ethanol+US probe
DGTS	0.33424	0.41780	Dichloromethane:methanol - Ethanol+US probe
PE	0.39299	0.42107	Ethanol - Ethanol+US probe
LPC	0.28147	0.42220	Ethanol - Ethanol+US probe
LPC	0.34000	0.42500	Chloroform:methanol - Ethanol
PG	0.37275	0.43009	Dichloromethane:methanol - Ethanol+US Bath
PE	0.37275	0.43009	Dichloromethane:ethanol - Ethanol
DGTS	0.31598	0.43088	Ethanol - Ethanol+US probe
PC	0.38137	0.44004	Chloroform:methanol - Dichloromethane:methanol
PE	0.35346	0.44183	Chloroform:methanol - Ethanol+US Bath
PG	0.41389	0.44346	Dichloromethane:ethanol - Ethanol
DGTS	0.30163	0.45245	Chloroform:methanol - Dichloromethane:ethanol
PE	0.35316	0.48158	Ethanol+US Bath - Ethanol+US probe
DGTS	0.29839	0.49732	Ethanol - Ethanol+US Bath
LPC	0.46749	0.50089	Chloroform:methanol - Ethanol+US Bath
PI	0.48041	0.55432	Dichloromethane:ethanol - Ethanol
SGDG	0.46215	0.57768	Chloroform:methanol - Ethanol+US Bath
PI	0.57747	0.61872	Ethanol+US Bath - Ethanol+US probe
DGDG	0.58731	0.62926	Chloroform:methanol - Dichloromethane:methanol
SGDG	0.59943	0.64224	Chloroform:methanol - Dichloromethane:methanol
PG	0.64897	0.64897	Chloroform:methanol - Dichloromethane:methanol
DGTS	0.61165	0.65534	Chloroform:methanol - Dichloromethane:methanol
MGDG	0.57532	0.66383	Chloroform:methanol - Ethanol+US probe
SGDG	0.57747	0.66631	Ethanol - Ethanol+US probe
DGTS	0.57747	0.66631	Dichloromethane:ethanol - Dichloromethane:methanol
DGTS	0.68719	0.68719	Chloroform:methanol - Ethanol+US probe
PC	0.65584	0.70268	Dichloromethane:ethanol - Ethanol
MGDG	0.79488	0.79488	Ethanol - Ethanol+US Bath
PC	0.79488	0.79488	Dichloromethane:methanol - Ethanol+US Bath
SGDG	0.82366	0.82366	Dichloromethane:methanol - Ethanol+US Bath
MGDG	0.79488	0.85166	Dichloromethane:ethanol - Dichloromethane:methanol
DGDG	0.85268	0.85268	Dichloromethane:ethanol - Ethanol
PE	0.87480	0.87480	Chloroform:methanol - Dichloromethane:methanol
PI	0.93720	0.93720	Chloroform:methanol - Dichloromethane:methanol
LPC	1.00000	1.00000	Dichloromethane:methanol - Ethanol

Supplementary Table S5. Main polar lipid species contributors for the PCA first dimension. Abbreviations: monogalactosyl diacylglycerol – MGDG, monogalactosyl monoacylglycerol – MGMG, sulfoquinovosyl diacylglycerol – SQDG, sulfoquinovosyl monoacylglycerol – SQMG, phosphatidylglycerol – PG, phosphatidylinositol – PI, monoacylglyceryl-O-4′-(N,N,N-trimethyl) homoserine – MGTS.

Label	Dim. 1 (%)
**SQDG321**	1.2793
**PI343**	0.8036
**PI342**	0.7991
**SQMG160**	0.7884
**PI301**	0.7745
**PI365**	0.2954
**MGMG161**	0.2593
**MGDG323**	0.2228
**MGDG385**	0.2069
**MGDG388**	0.1750
**PG362**	0.0698
**MGTS160**	0.0617
**MGMG140**	0.0509
**MGMG204**	0.0372
**MGMG160**	0.0182
**MGTS204**	0.0092
**SQDG302**	0.0002

Supplementary Table S6. Results of Kruskal-Wallis H test for statistically significant differences of polar lipid species between the groups. Abbreviations: monogalactosyl diacylglycerol – MGDG, monogalactosyl monoacylglycerol – MGMG, digalactosyl diacylglycerol – DGDG, digalactosyl monoacylglycerol – DGMG, sulfoquinovosyl diacylglycerol – SQDG, sulfoquinovosyl monoacylglycerol – SQMG, phosphatidylcholine – PC, lyso phosphatidylcholine – LPC, phosphatidylethanolamine – PE, phosphatidylglycerol – PG, phosphatidylinositol – PI, phosphoinositol ceramide – PI-Cer, diacylglyceryl-O-4′-(N,N,N-trimethyl) homoserine – DGTS, monoacylglyceryl-O-4′-(N,N,N-trimethyl) homoserine – MGTS.

Label	K.W	Significance	FDR
**PG365**	26.22	0.00008	0.00102
**PE322**	26.16	0.00008	0.00102
**PG322**	25.90	0.00009	0.00102
**PG342**	25.82	0.00010	0.00102
**PI321**	25.82	0.00010	0.00102
**PI342**	25.76	0.00010	0.00102

**DGDG321**	25.71	0.00010	0.00102
**DGDG342**	25.29	0.00012	0.00102
**PI322**	25.24	0.00012	0.00102
**PC364**	25.24	0.00013	0.00102
**PI301**	25.23	0.00013	0.00102
**DGDG341**	25.20	0.00013	0.00102
**PG341**	25.14	0.00013	0.00102
**PI343**	25.08	0.00013	0.00102
**PG366**	24.89	0.00015	0.00102
**PG345**	24.88	0.00015	0.00102
**SQDG341**	24.87	0.00015	0.00102
**PE4010**	24.60	0.00017	0.00102
**PE409**	24.59	0.00017	0.00102
**PC363**	24.57	0.00017	0.00102
**PG321**	24.55	0.00017	0.00102
**MGTS161**	24.46	0.00018	0.00102
**SQDG321**	24.17	0.00020	0.00102
**PE342**	24.16	0.00020	0.00102
**PC342**	24.14	0.00020	0.00102
**DGDG301**	24.03	0.00021	0.00102
**PI-Cerd181/140**	24.03	0.00021	0.00102
**SQDG342**	23.89	0.00023	0.00102
**DGDG343**	23.79	0.00024	0.00102
**PG343**	23.75	0.00024	0.00102
**DGDG365**	23.70	0.00025	0.00102
**PI-Cerd181/141**	23.62	0.00026	0.00102
**PC343**	23.59	0.00026	0.00102
**DGDG322**	23.42	0.00028	0.00107
**LPC160**	23.32	0.00029	0.00108
**MGMG205**	23.26	0.00030	0.00108
**SQDG322**	23.04	0.00033	0.00110
**PE366**	23.03	0.00033	0.00110
**PC300**	23.03	0.00033	0.00110
**DGDG345**	22.76	0.00038	0.00120
**MGTS204**	22.61	0.00040	0.00120
**MGMG160**	22.58	0.00041	0.00120
**PC322**	22.57	0.00041	0.00120
**MGTS205**	22.57	0.00041	0.00120
**PE365**	22.41	0.00044	0.00122
**SQDG301**	22.40	0.00044	0.00122
**PC344**	22.32	0.00045	0.00122
**MGMG140**	22.27	0.00046	0.00122
**DGDG366**	22.23	0.00047	0.00122
**PC323**	22.21	0.00048	0.00122
**SQDG302**	22.16	0.00049	0.00122
**PC362**	22.15	0.00049	0.00122
**MGTS160**	21.88	0.00055	0.00128
**MGMG204**	21.86	0.00056	0.00128

**SQDG311**	21.84	0.00056	0.00128
**LPC161**	21.83	0.00056	0.00128
**PC385**	21.76	0.00058	0.00128
**PC386**	21.76	0.00058	0.00128
**PC301**	21.74	0.00059	0.00128
**DGDG4010**	21.66	0.00061	0.00128
**SQMG160**	21.64	0.00061	0.00128
**PE386**	21.62	0.00062	0.00128
**SQDG281**	21.40	0.00068	0.00138
**PC365**	21.38	0.00069	0.00138
**PG301**	21.23	0.00073	0.00145
**SQDG365**	21.17	0.00075	0.00146
**PE321**	21.16	0.00076	0.00146
**PC387**	21.07	0.00078	0.00149
**DGDG344**	21.04	0.00079	0.00149
**LPC181**	21.00	0.00081	0.00149
**DGTS345**	20.75	0.00090	0.00164
**MGDG366**	20.68	0.00093	0.00166
**DGTS367**	20.54	0.00099	0.00172
**MGDG325**	20.54	0.00099	0.00172
**PC366**	20.44	0.00103	0.00178
**LPC182**	20.39	0.00106	0.00179
**LPC204**	20.27	0.00111	0.00186
**SQDG323**	20.15	0.00117	0.00194
**LPC140**	20.04	0.00123	0.00201
**LPC162**	20.00	0.00125	0.00202
**MGDG408**	19.92	0.00129	0.00205
**MGDG4010**	19.88	0.00131	0.00205
**DGTS388**	19.87	0.00132	0.00205
**LPC205**	19.66	0.00145	0.00221
**PC367**	19.63	0.00147	0.00221
**MGDG365**	19.62	0.00147	0.00221
**PC388**	19.59	0.00149	0.00221
**MGDG301**	19.56	0.00151	0.00221
**MGDG387**	19.54	0.00152	0.00221
**DGMG161**	19.35	0.00165	0.00237
**DGTS342**	19.30	0.00169	0.00239
**PE408**	19.17	0.00178	0.00250
**MGMG161**	19.05	0.00188	0.00261
**LPC183**	18.89	0.00201	0.00275
**MGDG386**	18.88	0.00203	0.00275
**DGTS4010**	18.78	0.00212	0.00284
**DGMG160**	18.69	0.00219	0.00292
**PC409**	18.57	0.00231	0.00304
**MGDG322**	18.39	0.00250	0.00325
**DGTS409**	18.19	0.00272	0.00351
**PC389**	18.09	0.00284	0.00362
**PI365**	18.00	0.00294	0.00372

**DGTS344**	17.61	0.00347	0.00435
**PE301**	17.50	0.00364	0.00451
**DGTS323**	17.25	0.00405	0.00498
**DGTS366**	16.97	0.00456	0.00555
**DGTS387**	16.83	0.00484	0.00583
**PE385**	16.70	0.00510	0.00609
**DGTS365**	16.16	0.00640	0.00753
**PG300**	16.15	0.00642	0.00753
**DGTS364**	15.95	0.00700	0.00813
**MGDG388**	15.84	0.00733	0.00844
**MGDG323**	15.82	0.00739	0.00844
**PC4010**	15.75	0.00759	0.00859
**DGDG367**	15.57	0.00820	0.00919
**DGTS301**	15.53	0.00834	0.00920
**DGTS386**	15.52	0.00835	0.00920
**MGDG342**	15.42	0.00871	0.00952
**PG362**	15.34	0.00899	0.00975
**MGDG345**	15.13	0.00981	0.01054
**DGTS322**	15.11	0.00992	0.01057
**MGDG321**	15.02	0.01026	0.01085
**DGTS385**	14.97	0.01047	0.01095
**MGDG385**	14.96	0.01052	0.01095
**DGTS343**	14.76	0.01142	0.01178
**DGTS341**	13.27	0.02098	0.02148
**DGDG387**	13.12	0.02226	0.02261
**DGTS321**	12.74	0.02592	0.02612
**DGDG325**	12.28	0.03116	0.03116
